# A Prospective International Multicentre Cohort Study of Intraoperative Heart Rate and Systolic Blood Pressure and Myocardial Injury After Noncardiac Surgery: Results of the VISION Study

**DOI:** 10.1213/ANE.0000000000002560

**Published:** 2017-10-26

**Authors:** Tom E. F. Abbott, Rupert M. Pearse, R. Andrew Archbold, Tahania Ahmad, Edyta Niebrzegowska, Andrew Wragg, Reitze N. Rodseth, Philip J. Devereaux, Gareth L. Ackland

**Affiliations:** From the * William Harvey Research Institute, Queen Mary University of London, London, United Kingdom; † Barts Health NHS Trust, London, United Kingdom; ‡ University of KwaZulu-Natal, Pietermaritzburg, South Africa; § Population Health Research Institute, McMaster University, Hamilton, Ontario, Canada.

## Abstract

**BACKGROUND:**

The association between intraoperative cardiovascular changes and perioperative myocardial injury has chiefly focused on hypotension during noncardiac surgery. However, the relative influence of blood pressure and heart rate (HR) remains unclear. We investigated both individual and codependent relationships among intraoperative HR, systolic blood pressure (SBP), and myocardial injury after noncardiac surgery (MINS).

**METHODS:**

Secondary analysis of the Vascular Events in Noncardiac Surgery Cohort Evaluation (VISION) study, a prospective international cohort study of noncardiac surgical patients. Multivariable logistic regression analysis tested for associations between intraoperative HR and/or SBP and MINS, defined by an elevated serum troponin T adjudicated as due to an ischemic etiology, within 30 days after surgery. Predefined thresholds for intraoperative HR and SBP were: maximum HR >100 beats or minimum HR <55 beats per minute (bpm); maximum SBP >160 mm Hg or minimum SBP <100 mm Hg. Secondary outcomes were myocardial infarction and mortality within 30 days after surgery.

**RESULTS:**

After excluding missing data, 1197 of 15,109 patients (7.9%) sustained MINS, 454 of 16,031 (2.8%) sustained myocardial infarction, and 315 of 16,061 patients (2.0%) died within 30 days after surgery. Maximum intraoperative HR >100 bpm was associated with MINS (odds ratio [OR], 1.27 [1.07–1.50]; *P* < .01), myocardial infarction (OR, 1.34 [1.05–1.70]; *P* = .02), and mortality (OR, 2.65 [2.06–3.41]; *P* < .01). Minimum SBP <100 mm Hg was associated with MINS (OR, 1.21 [1.05–1.39]; *P* = .01) and mortality (OR, 1.81 [1.39–2.37]; *P* < .01), but not myocardial infarction (OR, 1.21 [0.98–1.49]; *P* = .07). Maximum SBP >160 mm Hg was associated with MINS (OR, 1.16 [1.01–1.34]; *P* = .04) and myocardial infarction (OR, 1.34 [1.09–1.64]; *P* = .01) but, paradoxically, reduced mortality (OR, 0.76 [0.58–0.99]; *P* = .04). Minimum HR <55 bpm was associated with reduced MINS (OR, 0.70 [0.59–0.82]; *P* < .01), myocardial infarction (OR, 0.75 [0.58–0.97]; *P* = .03), and mortality (OR, 0.58 [0.41–0.81]; *P* < .01). Minimum SBP <100 mm Hg with maximum HR >100 bpm was more strongly associated with MINS (OR, 1.42 [1.15–1.76]; *P* < .01) compared with minimum SBP <100 mm Hg alone (OR, 1.20 [1.03–1.40]; *P* = .02).

**CONCLUSIONS:**

Intraoperative tachycardia and hypotension are associated with MINS. Further interventional research targeting HR/blood pressure is needed to define the optimum strategy to reduce MINS.

**KEY POINTS**

- **Question:** Are intraoperative heart rate and/or systolic blood pressure associated with myocardial injury after noncardiac surgery?
- **Findings:** Very high heart rate and very high or low systolic blood pressure during surgery are associated with increased risk of myocardial injury after noncardiac surgery.
- **Meaning:** Further targeted interventional studies using intraoperative heart rate and/or blood pressure thresholds that we have identified may help identify strategies to reduce perioperative cardiac complications.

One in 10 patients sustain myocardial injury after noncardiac surgery (MINS),^1^ characterized by a transient rise in serum troponin levels usually unaccompanied by any clinical or electrocardiographic signs/symptoms.^2^ Postoperative troponin elevation is strongly associated with death after surgery, which occurs in up to 1%–4% of 8 million surgical procedures performed in the United Kingdom each year.^1,3–5^ Although the etiology of MINS remains unclear,^6^ numerous retrospective studies implicate extreme intraoperative changes in blood pressure and/or heart rate (HR).^7,8^ However, few of these studies have used an objective, subclinical marker of MINS.

Tachycardia and hypotension, either separately or in combination, may provoke MINS, via a presumed mechanism of oxygen supply-demand-imbalance.^7–10^ Although preoperative resting HR is associated with postoperative MINS, it is uncertain if this relationship continues during surgery.^11^ Attempts to control elevated HR with β-blockers have consistently demonstrated a reduction in myocardial infarction; however, trials have also demonstrated an increase in mortality and stroke with perioperative β-blocker treatment.^12,13^ Moreover, the impact of the combination of intraoperative tachycardia and hypotension on MINS remains unclear. Similarly, how the duration of hemodynamic abnormalities influence the development of MINS is uncertain and under-investigated.^14–17^

In the largest prospective perioperative cohort of its kind, we tested whether high or low intraoperative HR or systolic blood pressure (SBP), in isolation or combination, were associated with MINS, myocardial infarction, or mortality within 30 days after noncardiac surgery in the Vascular Events in Noncardiac Surgery Cohort Evaluation (VISION) study cohort. In addition, we tested whether the duration of high or low HR/SBP was associated with MINS within 30 days of noncardiac surgery.

## METHODS

### Study Design

This was a secondary analysis of a prospective international observational cohort study, the VISION study.^1^ The methods have been published previously and the study was registered with clinicaltrials.gov (NCT00512109).^1,11,18,19^ This analysis was planned before taking custody of data; exposures and outcomes were defined a priori. Ethics committees or institutional review boards at each site reviewed and approved the protocol, and the research was consistent with the principles of the declaration of Helsinki. All participants or their designates provided written informed consent to take part in the study. Eight hospitals used deferred consent for patients who were unable to provide consent and had no next of kin available. Where it was not possible to approach the patient before surgery (eg, emergency surgery), they were approached for written consent within 24 hours after the procedure. This report follows the Strengthening the Reporting of Observational Studies in Epidemiology (STROBE) guidelines for observational cohort studies.^20^

### Participants

VISION study participants were aged 45 years or older, underwent noncardiac surgery under general or regional anesthesia, and stayed in hospital for at least 1 night. Patients were excluded if they refused to provide consent or if they had previously taken part in the VISION study.

### Data Collection

Data were collected prospectively by research personnel at each hospital before, during, and after surgery. There was a follow-up visit or telephone call at 30 days after surgery. Medical records were reviewed prospectively and data were transcribed to standardized case report forms.

### Exposure Variables

Clinical staff measured HR and blood pressure before and during surgery as part of routine medical care according to local practice. Blood pressure was most often measured using the oscillometric, noninvasive technique. During surgery, the highest and lowest single measurements of HR and SBP, duration of HR >100 bpm and <55 bpm, and duration of SBP >160 mm Hg and <100 mm Hg were recorded by reviewing the anesthetic charts or record. Duration was defined as the total time above or below the predefined thresholds during surgery, measured in minutes. Predefined and pragmatic thresholds for HR and blood pressure were chosen prospectively by consensus of VISION study investigators.

### Outcome Measures

The primary outcome measure was MINS, according to the VISION study definition: serum Troponin T (TnT) ≥0.03 ng/mL within 30 days after surgery, adjudicated as due to an ischemic pathology, which excludes nonischemic causes of transient troponin elevation.^19,21^ TnT was measured using a Roche 4th generation Elecsys assay. Blood was sampled between 6 and 12 hours after surgery was completed, and then again on postoperative days 1, 2, and 3. In addition, investigators were encouraged to take additional blood samples if participants experienced an ischemic symptom within the 30-day postoperative period. Since TnT ≥0.04 ng/mL was the accepted laboratory threshold at many hospitals when the study started, electrocardiograms were only routinely performed when troponin concentration reached this threshold. Echocardiograms were recommended in the absence of clinical signs or electrocardiographic evidence of myocardial ischemia. Patients with a serum troponin elevation <0.04 ng/mL were not investigated for evidence of myocardial ischemia. The secondary outcome was myocardial infarction within 30 days of surgery. Myocardial infarction was defined according to the third universal definition (serum troponin elevation in the presence of at least one of ischemic symptoms; the development of new or presumed new Q waves, ST segment or T wave changes, or left bundle branch block on the electrocardiogram; or the finding of a new or presumed new regional wall motion abnormality on echocardiography).^22^ The tertiary outcome measure was all-cause mortality within 30 days after surgery.

### Statistical Analysis

**Figure 1.**
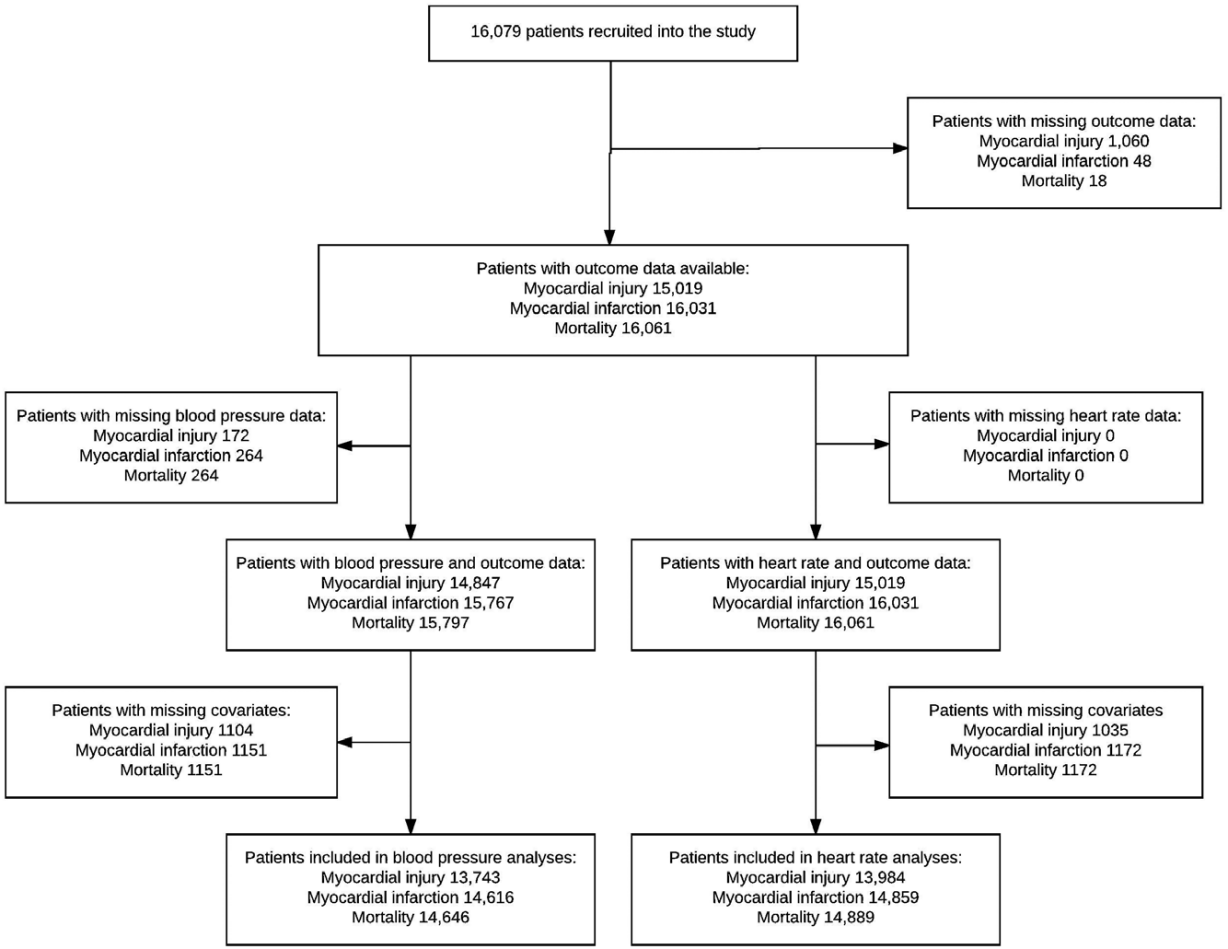
Patient flow diagram showing the number of cases included and excluded from each analysis.

We used SPSS (IBM, New York, NY) for the primary analysis, which we planned before taking custody of the data. Cases that were missing a record of highest or lowest intraoperative HR or SBP, or outcome data were excluded from respective analyses by list-wise deletion (Figure 1). We sorted and dichotomized the sample according to predefined thresholds for highest intraoperative HR (>100 bpm), lowest intraoperative HR (<55 bpm), highest intraoperative SBP (>160 mm Hg), and lowest intraoperative SBP (<100 mm Hg), and considered these as categorical variables. We presented demographic data stratified according to these groups. Continuous data that followed a normal distribution were presented as mean (standard deviation), continuous data that did not follow a normal distribution were presented as median (interquartile range), and binary categorical data as frequencies with percentages.

We used multivariable logistic regression analysis to test for associations between independent variables and MINS within 30 days after surgery. The reference groups were HR ≤100 bpm for highest HR, HR ≥55 bpm for lowest HR, SBP ≤160 mm Hg for highest systolic pressure, and SBP ≥100 mm Hg for lowest systolic pressure. Each multivariable model was adjusted for potentially confounding factors known to be associated with MINS, cardiovascular complications, or mortality in other perioperative research: age (45–64, 65–75, >75 years), current atrial fibrillation, diabetes, hypertension, heart failure, coronary artery disease, peripheral vascular disease, previous stroke or transient ischemic attack, estimated glomerular filtration rate (<30, 30–44, 45–60, >60 mL/min), chronic obstructive pulmonary disease, neurosurgery, major surgery, and urgent/emergency surgery were considered as categorical variables in the multivariable models.^1,23–25^ Full definitions of covariates are listed in Supplemental Digital Content, http://links.lww.com/AA/C70. These analyses were repeated for the secondary outcomes: mortality and myocardial infarction. The results of logistic regression analyses were presented as odds ratios (OR) with 95% confidence intervals. For the primary (MINS) analysis, the available sample size was 15,109. Given a type I error rate of 5% and a background incidence of MINS of 7.9%, we have >99% power to detect a 1.8% absolute difference in the incidence of MINS for participants with intraoperative HR >100 bpm. The minimum sample size required to detect an absolute difference of 1.8% in the primary outcome, assuming a type I error rate of 5% and power of 99%, is 4967 participants. Additional power calculations for the secondary and tertiary outcomes are in Supplemental Digital Content, http://links.lww.com/AA/C70.

We suspected that any relationship between duration of intraoperative HR >100/<55 bpm or SBP >160/<100 mm Hg and MINS would be nonlinear. Therefore, we stratified duration of intraoperative HR >100/<55 bpm or SBP >160/<100 mm Hg into quartiles and repeated the primary multivariable logistic regression analysis for quartiles of duration and repeated as for the primary analysis. Quartiles of duration were considered as ordered categorical variables. The reference categories were patients with “normal” HR or SBP, for example, in the analysis of duration of HR >100 bpm, the reference group was patients with HR ≤100 bpm. We undertook post hoc Bonferroni corrections to adjust for multiple comparisons. We were interested to see whether at least 1 episode (a single measurement) of “abnormal” HR and at least 1 episode of “abnormal” SBP during the same operative episode influenced the degree of association with MINS. We therefore undertook a planned sensitivity analysis to examine the relationship between MINS and combinations of abnormal HR and SBP during a single surgical procedure; because the data were not time-stamped, these were not necessarily concurrent/simultaneous episodes. We categorized the cohort according to combinations of highest/lowest HR and highest/lowest SBP: highest HR and highest SBP/highest HR and lowest SBP/highest SBP and lowest HR/lowest SBP and lowest HR. We repeated primary statistical analysis using these categorical variables.

### Sensitivity Analyses

To determine the influence of emergency surgery, we excluded all emergency cases and repeated the primary analyses. To determine the influence of atrial fibrillation, we repeated the primary HR analyses after excluding all cases with a previous history of atrial fibrillation. To determine the influence of HR modulating medications—β-adrenoceptor antagonists (β-blockers) and calcium channel blockers (diltiazem and verapamil)—we excluded patients who received a β-blocker and/or a calcium channel blocker within 24 hours before surgery and repeated the primary analysis of HR. In the primary analysis, the independent variables were high/low HR/SBP categorized according to 1 or more episodes above/below predefined binary thresholds. However, this did not take account of instances where a patient had both high and low HR/SBP episodes during surgery. Therefore, we undertook a post hoc sensitivity analysis. HR was categorized as follows: 55–100 bpm, minimum HR <55 bpm, maximum HR >100 bpm, minimum HR <55 bpm, and maximum HR >100 bpm. SBP was categorized as follows: 100–160 mm Hg, minimum SBP <100 mm Hg, maximum SBP >160 mm Hg, minimum SBP <100 mm Hg, and maximum SBP >160 mm Hg. We repeated the primary analysis to test for association between these 4-level HR/SBP variables and MINS.

## RESULTS

**Table 1.**
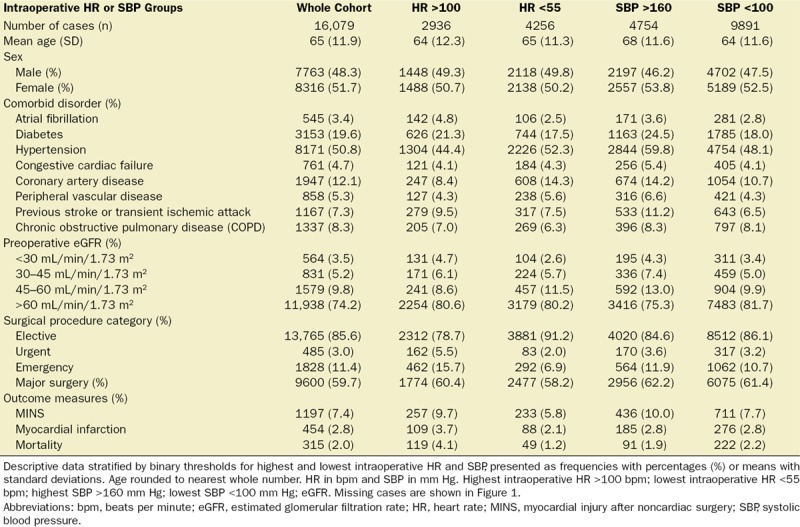
Baseline Patient Characteristics

A total of 16,079 patients were recruited to the VISION study from 12 hospitals in 8 countries between August 6, 2007 and January 11, 2011.^1^ A total of 1197 of 15,109 patients (7.9%) sustained MINS, 454 of 16,031 patients (2.8%) sustained myocardial infarction, and 315 of 16,061 patients (2.0%) died, within 30 days of surgery. Baseline characteristics and simple frequencies and proportions of the main outcomes, stratified by the exposures of interest, are presented in Table 1. Cases included in multivariable analyses are shown in Figure 1.

### Intraoperative HR

**Table 2.**
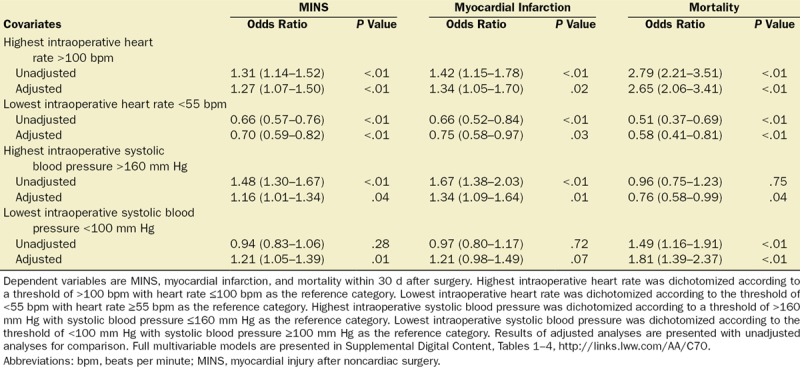
Summary Multivariable Logistic Regression Models for Highest and Lowest Intraoperative Heart Rate and Systolic Blood Pressure

**Figure 2.**
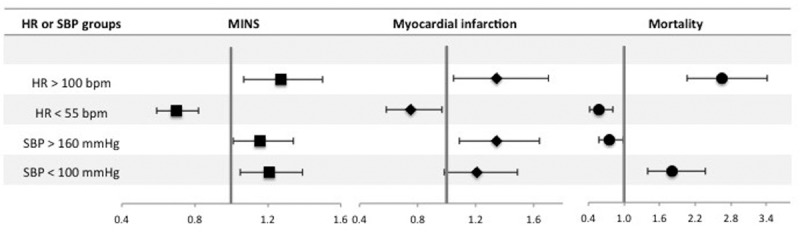
Forest plot summarizing multivariable logistic regression models for highest and lowest intraoperative heart rate (HR) and systolic blood pressure (SBP). Dependent variables are myocardial injury after noncardiac surgery (MINS), myocardial infarction, and mortality within 30 d after surgery. Highest intraoperative HR was dichotomized according to a threshold of >100 beats per minute (bpm) with HR ≤100 bpm as the reference category. Lowest intraoperative HR was dichotomized according to the threshold of <55 bpm with HR ≥55 bpm as the reference category. Highest intraoperative SBP was dichotomized according to a threshold of >160 mm Hg with SBP ≤160 mm Hg as the reference category. Lowest intraoperative SBP was dichotomized according to the threshold of <100 mm Hg with SBP ≥100 mm Hg as the reference category. The x-axis shows odds ratios and the error bars show 95% confidence intervals. Full multivariable models are presented in Supplemental Digital Content, Tables 1–4, http://links.lww.com/AA/C70.

**Figure 3.**
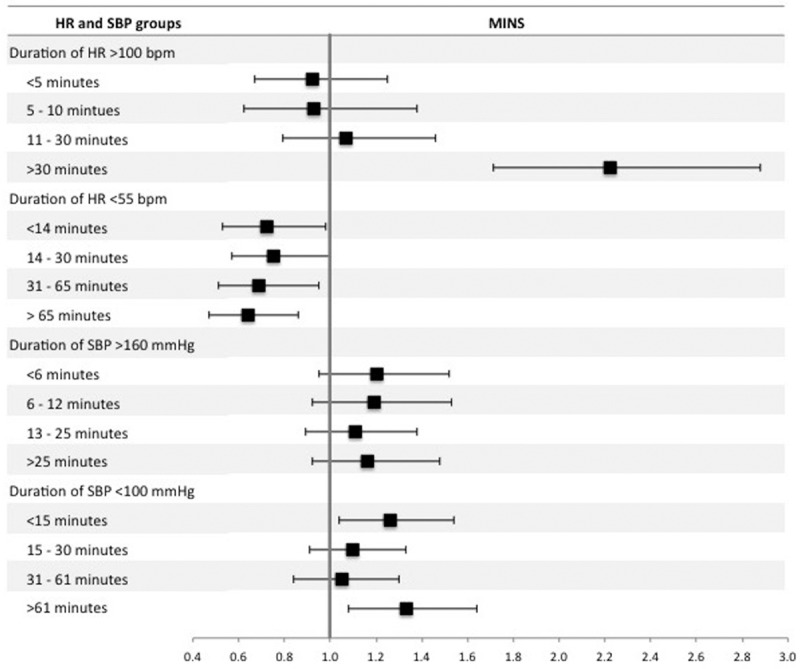
Forest plot summarizing multivariable logistic regression models for the duration of high/low intraoperative heart rate (HR) and systolic blood pressure (SBP). The dependent variable was myocardial injury after noncardiac surgery (MINS) within 30 d after surgery. There were 4 separate regression models for duration of intraoperative HR >100 beats per minute (bpm), intraoperative HR <55 bpm intraoperative SBP >160 mm Hg, and intraoperative SBP <100 mm Hg. For each model, duration was stratified into 4 approximately equal quartiles. The reference categories were patients with “normal” HR or SBP, for example in the analysis of duration of HR >100 bpm, the reference group was patients with HR ≤100 bpm. The x-axis shows odds ratios and the error bars show 95% confidence intervals. The full multivariable regression models are presented in Supplemental Digital Content, Tables 3, 4, 7, and 8, http://links.lww.com/AA/C70.

Highest intraoperative HR >100 bpm was associated with increased odds of MINS (OR, 1.27 [1.07–1.50]; *P* < .01), myocardial infarction (OR, 1.34 [1.05–1.70]; *P* = .02), and mortality (OR, 2.65 [2.06–3.41]; *P* < .01). Lowest intraoperative HR <55 bpm was associated with reduced odds of MINS (OR, 0.70 [0.59–0.82]; *P* < .01), myocardial infarction (OR, 0.75 [0.58–0.97]; *P* = .03), and mortality (OR, 0.58 [0.41–0.81]; *P* < .01) (Table 2, Figure 2; Supplemental Digital Content, Tables 1 and 2, http://links.lww.com/AA/C70). Duration of intraoperative HR >100 bpm for longer than 30 minutes was associated with MINS (OR, 2.22 [1.71–2.88]; *P* < .01) compared to participants with intraoperative hart rate ≤100 bpm throughout the procedure (Figure 3; Supplemental Digital Content, Table 3, http://links.lww.com/AA/C70). HR <55 bpm for any duration was associated with reduced odds of MINS and there was a trend toward reduced likelihood of MINS as duration of HR <55 bpm increased (Figure 3; Supplemental Digital Content, Table 4, http://links.lww.com/AA/C70).

### Intraoperative SBP

Highest intraoperative SBP >160 mm Hg was associated with increased odds of MINS (OR, 1.16 [1.01–1.34]; *P* = .04), myocardial infarction (OR, 1.34 [1.09–1.64]; *P* = .01), and reduced odds of mortality (OR, 0.76 [0.58–0.99]; *P* = .04). Lowest intraoperative SBP <100 mm Hg was associated with increased odds of MINS (OR, 1.21 [1.05–1.39]; *P* = .01) and mortality (OR, 1.81 [1.39–2.37]; *P* < .01), but was not associated with myocardial infarction (OR, 1.21 [0.98–1.49]; *P* = .07) (Table 2, Figure 2; Supplemental Digital Content, Tables 5 and 6, http://links.lww.com/AA/C70). Duration of SBP >160 mm Hg was not associated with MINS (Figure 3; Supplemental Digital Content, Table 7, http://links.lww.com/AA/C70). In comparison, duration of SBP <100 mm Hg for <15 or >61 minutes was associated with MINS (OR, 1.26 [1.04–1.54]; *P* = .02 and OR, 1.33 [1.08–1.64]; *P* < .01, respectively) (Figure 3; Supplemental Digital Content, Table 8, http://links.lww.com/AA/C70).

### Intraoperative HR and SBP

**Figure 4.**
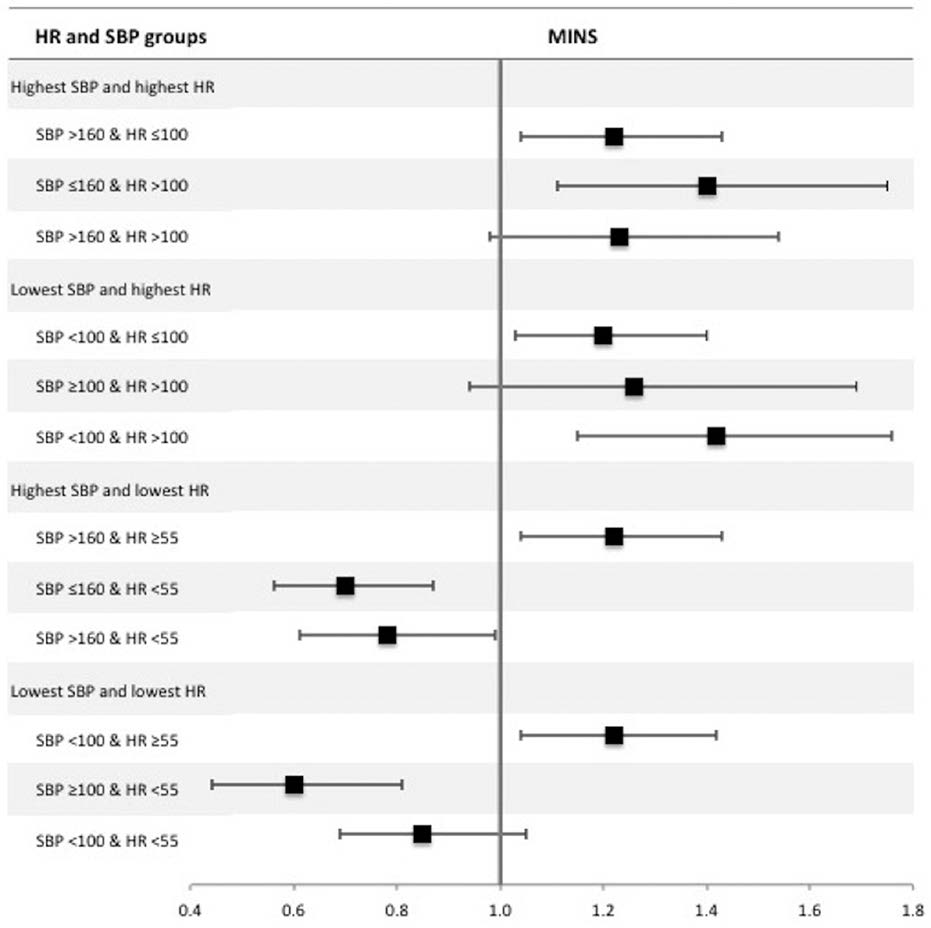
Forest plot summarizing multivariable logistic regression models for combinations of highest/lowest intraoperative systolic blood pressure (SBP) and heart rate (HR). The dependent variable was myocardial injury after noncardiac surgery (MINS) within 30 d after surgery. The sample was categorized according to highest intraoperative SBP >160 mm Hg, lowest intraoperative SBP <100 mm Hg, highest intraoperative HR >100 beats per minute (bpm), and lowest intraoperative HR <55 bpm. For highest SBP and HR, the reference group was SBP ≤160 and HR ≤100; for lowest SBP and highest HR, the reference group was SBP ≥100 and HR ≤100; for highest SBP and lowest HR, the reference group was SBP ≤160 and HR ≥55; and for lowest SBP and lowest HR, the reference group was SBP ≥100 and HR ≥55. The x-axis shows odds ratios and the error bars show 95% confidence intervals. The results presented are summaries of adjusted analyses (as per the primary analysis). Full multivariable models are presented in Supplemental Digital Content, Tables 9–12, http://links.lww.com/AA/C70.

Combinations of highest/lowest HR and SBP were categorized as follows: highest HR and highest SBP/highest HR and lowest SBP/highest SBP and lowest HR/lowest SBP and lowest HR. The association between HR and MINS was modified by SBP and is shown in Figure 4 and Supplemental Digital Content, Tables 9–12, http://links.lww.com/AA/C70. The incidence of MINS in patients with hypotension (SBP <100 mm Hg) and tachycardia (HR >100 bpm) was 176 of 1906 (9.2%) and had higher odds of MINS (OR, 1.42 [1.15–1.76]; *P* < .01), compared to patients with hypotension in the absence of tachycardia (499 of 6632 [7.5%]; OR, 1.20 [1.03–1.40]; *P* = .02) or patients with tachycardia in the absence of hypotension (76 of 736 [10.3%]; OR, 1.26 [0.94–1.69]; *P* = .13), where the reference group was patients without hypotension or tachycardia. Patients with hypertension (SBP >160 mm Hg) without bradycardia (HR <55 bpm) were at increased risk of MINS (326 of 2802 [11.6%]; OR, 1.22 [1.04–1.43]; *P* = .02). However, bradycardia was associated with less risk of MINS, regardless of highest SBP (Figure 4). A similar result was seen for hypotension with and without bradycardia (Figure 4). On post hoc testing, there was little evidence of statistical interaction (effect modification) between HR and blood pressure, except between elevated systolic pressure and elevated HR, where the association between elevated systolic pressure and MINS was increased in the presence of HR >100 bpm.

### Sensitivity Analyses

When we repeated the primary analyses excluding 1828 participants undergoing emergency surgery, our results were similar (Supplemental Digital Content, Tables 13–16, http://links.lww.com/AA/C70). When we repeated the primary HR analysis excluding 2727 participants who received either a β-blocker or rate-limiting calcium channel blocker within 24 hours before surgery, our results were very similar (Supplemental Digital Content, Tables 17 and 18, http://links.lww.com/AA/C70). However, the association between the lowest intraoperative HR <55 bpm and reduced mortality was no longer statistically significant (OR, 0.71 [0.49–1.03]; *P* = .07). When we repeated the primary HR analysis excluding 545 participants with preexisting atrial fibrillation, our results were very similar (Supplemental Digital Content, Tables 19 and 20, http://links.lww.com/AA/C70). However, the association between highest intraoperative HR >100 bpm was only a trend (OR, 1.29 [0.99–1.67]; *P* = .06). It is possible that a patient could have episodes of both high HR and low HR during the surgical procedure. The same is true for SBP. We undertook a post hoc analysis that categorized episodes of high/low HR/SBP into two 4-level categorical variables (HR: 55–100 bpm/<55 bpm/>100 bpm/<55 and >100 bpm; and SBP: 100–160 mm Hg/<100 mm Hg/>160 mm Hg/<100 and >160 mm Hg), shown in Supplemental Digital Content, Table 21, http://links.lww.com/AA/C70. The results were similar to the primary analysis (Supplemental Digital Content, Tables 22 and 23, http://links.lww.com/AA/C70), except that the association between maximum SBP >160 mm Hg and MINS was no longer statistically significant (OR, 1.22 [0.97–1.52]; *P* = .08). The combination of minimum SBP <100 mm Hg and maximum SBP >160 mm Hg was associated with MINS (OR, 1.42 [1.16–1.75]; *P* < .01). However, the combination of minimum HR <55 bpm and maximum HR >100 bpm was not associated with MINS (OR, 0.70 [0.44–1.13]; *P* = .15). To account for possible increased type I error associated with multiple comparisons in the analysis of duration of high/low HR/SBP quartiles, we undertook Bonferroni corrections. The results remained similar in that the longest durations of SBP <100 mm Hg (>61 minutes), HR >100 bpm (>30 minutes), and HR <55 bpm (>55 minutes) remained associated with MINS. However, the associations between intermediate durations and MINS were no longer statistically significant.

## DISCUSSION

The principal finding of this analysis is that intraoperative tachycardia and hypotension are independently associated with MINS and mortality. For the first time, we demonstrate the effect of combinations of intraoperative HR and SBP. Our results suggest that the association between low SBP and MINS is increased if elevated HR occurred during the procedure and reduced if low HR occurred during the procedure. Prolonged durations of HR >100 bpm and SBP <100 mm Hg were associated with increased risk of MINS. Furthermore, minimum intraoperative HR <55 bpm was associated with reduced risk of MINS and mortality.

Our results are consistent with studies that demonstrate association between intraoperative hypotension and postoperative adverse events after noncardiac surgery.^7,8,12,15,26,27^ However, unlike our study, these were small and few used objective biomarkers as outcome measures. This is the first study to identify relationships between high intraoperative HR/SBP and increased risk of MINS, and low intraoperative HR and reduced risk of MINS. While the observational nature of our data do not allow us to infer causal relationships, it is reasonable to hypothesize that active avoidance of either very high HR or very high/low SBPs during surgery may be clinically beneficial. Data from clinical trials suggest that treatment with β-blockers (which lower HR) can reduce the risk of myocardial infarction after noncardiac surgery, but at the expense of larger increases in the risk of mortality and stroke.^12,28^ However, it is difficult to disentangle the effect of HR from the effect of β-blockers and the degree of interaction between these variables. In our study, 2727 (16.9%) patients received a β-blocker or negatively chronotropic calcium channel blocker within 24 hours before surgery. We repeated the primary analysis after removing these cases and found that the associations between maximum intraoperative HR and all the outcomes, and between minimum intraoperative HR and MINS/myocardial infarction, were unchanged. However, the negative association between minimum intraoperative HR <55 bpm and mortality was no longer statistically significant. In other words, the “protective” association between low HR and reduced risk of mortality was lost in patients not receiving a β-blocker or calcium channel antagonist. We previously made a similar observation for low preoperative HR, which suggests confounding by rate-controlling medication.^11^ However, we are unable to infer a causative relationship due to the observational nature of our data. This contrasts with the results of previous clinical trials where HR lowering medication was associated with increased mortality.^12^ Further research is needed to investigate potential mechanisms underlying this observation, in addition to trials targeting HR control while avoiding hypotension.^29^

Our data suggest that very high or very low intraoperative HR and blood pressure are key perioperative factors that, alone or in tandem, may contribute to MINS. The potential for preoperative identification or treatment of these patients needs further research.^30,31^ The pathophysiological mechanism of MINS is unclear. It may be caused by extended periods of myocardial ischemia as a result of oxygen supply–demand imbalance in cardiac muscle, the proposed mechanism of type 2 myocardial infarction.^9,32^ In the context of this model, intraoperative hypotension or tachycardia may reduce myocardial perfusion pressure, leading to reduced myocardial oxygen supply. Similarly, elevated systolic pressure increases end-systolic stress, leading to increased myocardial oxygen demand.^33^ Our results support this theory; we observed increasing risk of MINS as the duration of tachycardia or hypotension increased. This is consistent with animal studies of tachycardia induced subendocardial myocardial necrosis, where the duration of ischemia was correlated with degree of necrosis.^34^ In addition, we observed that patients with very high and very low blood pressure during surgery were at greater risk of MINS than either high or low blood pressure alone. While we adjusted our analysis for multiple perioperative factors, our findings may reflect unmeasured confounding and cannot exclude that tachycardia/hypertension/hypotension are merely markers of other conditions or treatment interventions that may promote MINS.^35–39^

A strength of this analysis is the large sample size derived from multiple centers in multiple countries, giving robust external validity and making our results generalizable to the vast majority of patients undergoing noncardiac surgery. The primary outcome was an objective biomarker, rather than a potentially subjective, clinically defined outcome. The use of troponin is important because more than 4 of 5 patients with MINS are asymptomatic, while only one-third have electrocardiographic or echocardiographic evidence of ischemia.^19^ The detailed nature of the VISION database allowed us to adjust our analysis for confounding factors using multivariable regression modeling, including preexisting atrial fibrillation.

Our analyses also have some important limitations. We cannot exclude unmeasured confounding, for example, the use of intraoperative cardiac medication, the presence of cardiac pacemakers, and the incidence of preoperative troponin elevation are all unknown, although we expect the frequency of these to be low.^40,41^ Similarly, we did not routinely seek evidence of myocardial ischemia in patients with troponin elevation <0.04 ng/mL, which may underestimate the incidence of myocardial ischemia. Another potentially confounding factor is preexisting atrial fibrillation, which we corrected for by inclusion in the multivariable models and through sensitivity analysis. We performed additional sensitivity analyses that excluded cases of emergency surgery, and patients receiving preoperative β-blockers or rate-limiting calcium channel antagonists within 24 hours before surgery. However, in the case of rate-limiting medication, it is possible that the sensitivity analysis included patients who stopped taking these medications, with a variety of half-lives, >24 hours before surgery. It is possible that the type of anesthesia (volatile, intravenous, balanced, or regional techniques) could have confounded our results; however, these data were not available for this secondary analysis, so we were unable to undertake a post hoc sensitivity analysis on this basis. Continuous measurements of intraoperative HR and blood pressure were not recorded as part of this study; therefore our analysis was limited to summary data for intraoperative HR and blood pressure, which included predefined thresholds. Similarly, it is unclear why SBP <100 mm Hg for between 15 and 60 minutes was not associated with MINS. Future computational research using continuously recorded HR or blood pressure data should be considered. We could only adjust the analysis for the absolute duration of intraoperative tachycardia/hypotension rather than expressing this as a proportion of length of surgery. We undertook a planned sensitivity analysis to examine association between combinations of abnormal intraoperative HR and SBP and MINS, and a post hoc analysis of combinations of very high and very low blood pressure/HR during surgery and MINS. However, because our data did not include the exact timing of high/low HR/SBP, these combinations were not necessarily concurrent/simultaneous episodes. Our data suggest that future research to examine contemporaneous episodes of abnormal HR and SBP is warranted, particularly the combination of tachycardia and hypotension.

Very high HR and very high or low SBP during surgery are associated with increased risk of MINS. The duration of low intraoperative blood pressure and high HR are additional etiological factors for MINS. Further targeted interventional studies using intraoperative HR and/or blood pressure thresholds that we have identified may help identify strategies to reduce perioperative cardiac complications.

## DISCLOSURES

**Name:** Tom E. F. Abbott, MRCP.

**Contribution:** This author helped design the analysis plan, perform the data analysis, draft the manuscript, and approve the final version.

**Conflicts of Interest:** None.

**Name:** Rupert M. Pearse, MD.

**Contribution:** This author helped design the analysis plan, provide advice on the data analysis, draft the manuscript, and approve the final version.

**Conflicts of Interest:** R. M. Pearse holds research grants, and has given lectures and/or performed consultancy work for Nestle Health Sciences, BBraun, Medtronic, Glaxo Smithkline, and Edwards Lifesciences, and is a member of the associate editorial board of the *British Journal of Anaesthesia*.

**Name:** R. Andrew Archbold, MD.

**Contribution:** This author helped design the analysis plan, critically review the manuscript, and approve the final version.

**Conflicts of Interest:** None.

**Name:** Tahania Ahmad, MPH.

**Contribution:** This author helped analyze the data, critically review the manuscript, and approve the final version.

**Conflicts of Interest:** None.

**Name:** Edyta Niebrzegowska, MSc.

**Contribution:** This author helped critically review the manuscript and approve the final version.

**Conflicts of Interest:** None.

**Name:** Andrew Wragg, FRCP.

**Contribution:** This author helped critically review the manuscript and approve the final version.

**Conflicts of Interest:** None.

**Name:** Reitze N. Rodseth, PhD.

**Contribution:** This author helped design the analysis plan, critically review the manuscript, and approve the final version.

**Conflicts of Interest:** None.

**Name:** Philip J. Devereaux, PhD.

**Contribution:** This author helped design the analysis plan, provide advice on the data analysis, critically review the manuscript, and approve the final version.

**Conflicts of Interest:** P. J. Devereaux acts as a guarantor of the data and affirms the manuscript is an honest, accurate, and transparent account of the secondary analysis being reported. He has also received other funding from Roche Diagnostics and Abbott Diagnostics for investigator initiated studies.

**Name:** Gareth L. Ackland, PhD.

**Contribution:** This author helped design the analysis plan, provide advice on the data analysis, draft the manuscript, and approve the final version.

**Conflicts of Interest:** G. L. Ackland is a member of the associate editorial board of Intensive Care Medicine Experimental. This author acts as a guarantor of the data and affirms the manuscript is an honest, accurate, and transparent account of the secondary analysis being reported.

**This manuscript was handled by:** Richard C. Prielipp, MD.

## Supplementary Material
