# Supplementary material for: A Prospective International Multicentre Cohort Study of Intraoperative Heart Rate and Systolic Blood Pressure and Myocardial Injury After Noncardiac Surgery: Results of the VISION Study
Source: Anesth Analg. 2017 Oct 26;126(6):1936–45. doi: 10.1213/ANE.0000000000002560 (PMC5815500; doi:10.1213/ANE.0000000000002560)
Supplement: Supplementary file 1 [file ane-126-1936-s001.docx]

**A prospective international multi-centre cohort study of intraoperative heart rate and systolic blood pressure, and myocardial injury after non-cardiac surgery: results of the VISION study**

**T. E. F. Abbott, MRCP;^1^ R. M. Pearse, MD;^1^ R. A. Archbold, MD;^2^ T. Ahmad,^1^ E. Niebrzegowska, MSc;^2^ A. Wragg, FRCP;^2^ R.N. Rodseth, PhD;^3^ P. J. Devereaux, PhD;^4^ and G. L. Ackland, PhD.^1^**

**SUPPLEMENTARY FILE**

1. *William Harvey Research Institute, Queen Mary University of London, UK*
2. *Barts Health NHS Trust, London, UK*
3. *University of KwaZulu-Natal, Pietermaritzburg, 3200, South Africa*
4. *Population Health Research Institute, McMaster University, Canada*

Correspondence to:

Gareth Ackland

Translational Medicine and Therapeutics

William Harvey Research Institute

Queen Mary University of London

John Vane Science Centre

London, EC1M 6BQ

United Kingdom

e-mail: g.ackland@qmul.ac.uk

Tel: +44 20 3594 0351

**Preoperative patient characteristics and types of surgery**

1. Age – Patient age in years

2. Sex – Male or female.

3. Atrial fibrillation – a patient with a current history of atrial fibrillation

4. History of diabetes – Patient states that they have been diagnosed with diabetes or a physician has previously recorded that the patient has diabetes. Includes current gestational, but not past gestational diabetes that has resolved.

5. History of hypertension – A physician diagnosis of hypertension.

6. History of coronary artery disease – A current or prior history of any one of the following: i. angina; ii. myocardial infarction or acute coronary syndrome; iii. a segmental cardiac wall motion abnormality on echocardiography or a segmental fixed defect on radionuclide imaging; iv. a positive radionuclide exercise, echocardiographic exercise, or pharmacological cardiovascular stress test demonstrating cardiac ischemia; v. coronary angiographic or CT coronary angiographic evidence of atherosclerotic stenosis ≥50% of the diameter of any coronary artery; vi. ECG with pathological Q waves in two contiguous leads.

7. History of peripheral vascular disease – A physician diagnosis of a current or prior history of: intermittent claudication, vascular surgery for atherosclerotic disease, an ankle/arm systolic blood pressure ratio ≤ 0.90 in either leg at rest, or angiographic or Doppler study demonstrating ≥ 70% stenosis in a non-cardiac artery.

8. History of stroke or transient ischaemic attack – A physician diagnosis of stroke, CT or MRI evidence of a prior stroke, or physician diagnosis of a prior transient ischaemic attack (TIA).

9. History of chronic obstructive pulmonary disease (COPD) – If the chart or a physician has ever indicated that a patient has chronic bronchitis, accept it has a patient having COPD. If there is no mention of this but the patient tells you they have had daily production of sputum for at least 3 months in 2 consecutive years then they should be marked as having COPD. Likewise if a physician has ever indicated that a patient has emphysema or if a patient’s Pulmonary Function Tests (PFT) state a fixed or irreversible airflow limitation and/or emphysema then they should be marked as having COPD.

10. Preoperative estimated glomerular filtration rate (eGFR) rate as categorical variables (i.e., eGFR <30 ml/minute/1.73m^2^ or receiving dialysis; eGFR of 30 to 44 ml/minute/1.73m^2^; eGFR of 45 to 59 ml/minute/1.73m^2^; and the reference group eGFR of ≥60 ml/minute/1.73m^2^).

11. Urgency of surgery – Emergency surgery was surgery that occurred <24 hours after a patient developed an acute event that led to the need for surgery, and urgent surgery was surgery that occurred 24-72 hours after a patient developed an acute event that led to the need for surgery. Elective surgery was planned greater than 72 hours in advance.

**Ischemic symptoms and signs and electrocardiography findings**

1. Ischemic symptoms/signs included any of the following: chest discomfort, arm discomfort, neck discomfort, jaw discomfort, shortness of breath, or pulmonary oedema. These ischemic symptoms or signs had to have occurred within 24 hours of an elevated troponin measurement.

2. Ischemic electrocardiography findings included any of the following:

i. development of pathologic Q waves in any two contiguous leads that were ≥30 milliseconds; ii. development of left bundle branch block (LBBB); or

iii. development of ST segment elevation (≥2 mm in leads V_1_, V_2_, or V_3_ OR ≥1 mm in the other leads), ST segment depression (≥1 mm), or symmetric inversion of T waves ≥1 mm in at least two contiguous leads.

ST segment elevation, ST segment depression, and LBBB had to have occurred within 3 days of an elevated troponin measurement, and symmetric T wave inversion had to have occurred within 5 days of an elevated troponin measurement.

**Additional power calculations for mortality and myocardial infarction outcomes.**

Assuming a type I error rate of 5% and a total sample of 15,019, we have 99% power to detect an absolute difference of 2.1% (2.0% vs 4.1%) in the incidence of mortality for participants with intraoperative heart rate <100bpm and 99% power to detect an absolute difference of 0.9% (2.8% vs 3.7%) in the incidence of myocardial infarction. The minimum sample sizes required to detect these differences would be 1,639 participants for mortality and 8,082 participants for myocardial infarction.

**Supplementary table 1. Full adjusted logistic regression model for highest intraoperative heart rate**

**Multivariable logistic regression analysis. Dependent variables are myocardial injury, myocardial infarction and mortality within 30 days of surgery. Intraoperative heart rate was dichotomised according to a threshold of >100 beat per minute (bpm). Heart rate ≤100 bpm was the reference category. Estimated glomerular filtration rate (eGFR).**

|  | **Myocardial Injury** | | **Myocardial Infarction** | | **Mortality** | |
| --- | --- | --- | --- | --- | --- | --- |
| **Covariates** | **odds ratio** | **p-value** | **odds ratio** | **p-value** | **odds ratio** | **p-value** |
| Age (years) |  |  |  |  |  |  |
| 45-64 (reference) | - | - | - | - | - | - |
| 65-75 | 1.09 (0.91-1.31) | 0.39 | 1.17 (0.88-1.55) | 0.29 | 1.61 (1.15-2.25) | <0.01 |
| >75 | 2.06 (1.72-2.45) | <0.01 | 1.87 (1.43-2.45) | <0.01 | 2.35 (1.69-3.27) | <0.01 |
| Male sex | 1.37 (1.20-1.57) | <0.01 | 1.02 (0.83-1.26) | 0.83 | 1.18 (0.92-1.51) | 0.19 |
| History of atrial fibrillation | 1.58 (1.22-2.06) | <0.01 | 1.33 (0.93-1.90) | 0.12 | 1.00 (0.62-1.62) | 0.99 |
| History of diabetes | 1.40 (1.20-1.63) | <0.01 | 1.22 (0.98-1.53) | 0.08 | 1.02 (0.77-1.37) | 0.88 |
| History of hypertension | 1.31 (1.12-1.54) | <0.01 | 1.39 (1.08-1.77) | 0.01 | 0.99 (0.75-1.30) | 0.92 |
| History of heart failure | 1.60 (1.27-2.00) | <0.01 | 1.70 (1.27-2.30) | <0.01 | 1.45 (0.97-2.19) | 0.07 |
| History of coronary artery disease | 1.45 (1.23-1.72) | <0.01 | 2.21 (1.76-2.78) | <0.01 | 0.96 (0.69-1.34) | 0.81 |
| History of peripheral vascular disease | 2.17 (1.77-2.65) | <0.01 | 2.13 (1.62-2.81) | <0.01 | 1.84 (1.27-2.65) | <0.01 |
| History of stroke or transient ischaemic attack | 1.43 (1.18-1.74) | <0.01 | 1.11 (0.83-1.48) | 0.47 | 1.40 (1.00-1.96) | 0.05 |
| History of chronic obstructive pulmonary disease | 1.22 (1.00-1.50) | 0.05 | 1.15 (0.86-1.53) | 0.35 | 2.09 (1.52-2.86) | <0.01 |
| Preoperative eGFR (ml/min) |  |  |  |  |  |  |
| <30 | 10.76 (8.72-13.29) | <0.01 | 4.01 (2.98-5.38) | <0.01 | 3.03 (2.07-4.42) | <0.01 |
| 30-44 | 2.50 (2.02-3.10) | <0.01 | 1.72 (1.25-2.37) | <0.01 | 1.65 (1.11-2.44) | 0.01 |
| 45-60 | 1.68 (1.39-2.04) | <0.01 | 1.42 (1.06-1.90) | 0.02 | 0.99 (0.67-1.47) | 0.97 |
| >60 (reference) | - | - | - | - | - | - |
| Neurosurgery | 1.12 (0.85-1.48) | 0.42 | 0.57 (0.34-0.97) | 0.04 | 1.70 (1.10-2.63) | 0.02 |
| Urgent or emergency surgery | 1.93 (1.64-2.27) | <0.01 | 2.29 (1.83-2.87) | <0.01 | 3.46 (2.69-4.44) | <0.01 |
| Major surgery | 1.66 (1.42-1.94) | <0.01 | 2.21 (1.72-2.83) | <0.01 | 1.57 (1.18-2.10) | <0.01 |
| Maximum intraoperative heart rate (bpm) |  |  |  |  |  |  |
| >100 | 1.27 (1.07-1.50) | <0.01 | 1.34 (1.05-1.70) | 0.02 | 2.65 (2.06-3.41) | <0.01 |
|  |  |  |  |  |  |  |

**Supplementary table 2. Full adjusted logistic regression model for lowest intraoperative heart rate**

**Multivariable logistic regression analysis. Dependent variables are myocardial injury, myocardial infarction and mortality within 30 days of surgery. Intraoperative heart rate was dichotomised according to a threshold of <55 beats per minute (bpm). Heart rate ≥55 bpm was the reference category. Estimated glomerular filtration rate (eGFR).**

|  | **Myocardial Injury** | | **Myocardial Infarction** | | **Mortality** | |
| --- | --- | --- | --- | --- | --- | --- |
| **Covariates** | **odds ratio** | **p-value** | **odds ratio** | **p-value** | **odds ratio** | **p-value** |
| Age (years) |  |  |  |  |  |  |
| 45-64 (reference) | - | - | - | - | - | - |
| 65-75 | 1.09 (0.91-1.31) | 0.35 | 1.17 (0.88-1.55) | 0.29 | 1.54 (1.10-2.15) | 0.01 |
| >75 | 2.05 (1.72-2.44) | <0.01 | 1.86 (1.42-2.43) | <0.01 | 2.22 (1.60-3.09) | <0.01 |
| Male sex | 1.39 (1.21-1.59) | <0.01 | 1.03 (0.84-1.27) | 0.75 | 1.22 (0.96-1.56) | 0.11 |
| History of atrial fibrillation | 1.59 (1.23-2.07) | <0.01 | 1.35 (0.95-1.93) | 0.10 | 1.11 (0.69-1.78) | 0.66 |
| History of diabetes | 1.39 (1.20-1.62) | <0.01 | 1.22 (0.98-1.53) | 0.08 | 1.03 (0.77-1.38) | 0.83 |
| History of hypertension | 1.31 (1.12-1.53) | <0.01 | 1.38 (1.08-1.76) | 0.01 | 0.96 (0.73-1.26) | 0.75 |
| History of heart failure | 1.58 (1.25-1.98) | <0.01 | 1.68 (1.24-2.26) | <0.01 | 1.37 (0.91-2.06) | 0.13 |
| History of coronary artery disease | 1.46 (1.23-1.73) | <0.01 | 2.20 (1.75-2.77) | <0.01 | 0.90 (0.65-1.26) | 0.55 |
| History of peripheral vascular disease | 2.17 (1.77-2.65) | <0.01 | 2.1 1 (1.60 -2.79) | <0.01 | 1.79 (1.24-2.57) | <0.01 |
| History of stroke or transient ischaemic attack | 1.46 (1.20-1.77) | <0.01 | 1.13 (0.85-1.51) | 0.41 | 1.52 (1.09-2.12) | 0.01 |
| History of chronic obstructive pulmonary disease | 1.19 (0.97-1.45) | 0.10 | 1.12 (0.84-1.50) | 0.44 | 1.98 (1.45-2.70) | <0.01 |
| Preoperative eGFR (ml/min) |  |  |  |  |  |  |
| <30 | 10.71 (8.67-13.23) | <0.01 | 4.01 (2.98-5.39) | <0.01 | 3.15 (2.16-4.59) | <0.01 |
| 30-44 | 2.54 (2.05-3.15) | <0.01 | 1.74 (1.26-2.40) | <0.01 | 1.71 (1.16-2.53) | <0.01 |
| 45-60 | 1.69 (1.40-2.05) | <0.01 | 1.43 (1.07-1.91) | 0.02 | 0.98 (0.66-1.45) | 0.91 |
| >60 (reference) | - | - | - | - | - | - |
| Neurosurgery | 1.17 (0.88-1.54) | 0.28 | 0.59 (0.35-0.99) | 0.05 | 1.72 (1.11-2.66) | 0.02 |
| Urgent or emergency surgery | 1.89 (1.61-2.22) | <0.01 | 2.30 (1.83-2.88) | <0.01 | 3.71 (2.89-4.75) | <0.01 |
| Major surgery | 1.66 (1.42-1.93) | <0.01 | 2.20 (1.71-2.81) | <0.01 | 1.60 (1.20-2.14) | <0.01 |
| Minimum intraoperative heart rate (bpm) |  |  |  |  |  |  |
| <55 | 0.70 (0.59-0.82) | <0.01 | 0.75 (0.58-0.97) | 0.03 | 0.58 (0.41-0.81) | <0.01 |
|  |  |  |  |  |  |  |

**Supplementary table 3. Full adjusted logistic regression model for duration of intraoperative heart rate >100 beats per minute.**

**Multivariable logistic regression analysis. Dependent variables is myocardial injury within 30 days of surgery. Duration of heart rate >100 beats per minute (bpm) was stratified into quartiles. The reference group was participants with heart rate** ≤ **100 beats per minute. Estimated glomerular filtration rate (eGFR). A post-hoc Bonferroni correction was made for the four categories of duration, where p-values should be less than 0.05/4 = 0.0125 in order to be statistically significant (indicated in italics).**

| **Covariates** | **odds ratio** | **p-value** |
| --- | --- | --- |
| Age |  |  |
| 45-64 | ref | ref |
| 65-75 | 1.10 (0.91-1.32) | 0.33 |
| >75 | 2.09 (1.75-2.49) | <0.01 |
| Male sex | 1.37 (1.19-1.57) | <0.01 |
| Comorbid disease |  |  |
| Existing Atrial Fibrillation | 1.54 (1.19-2.01) | <0.01 |
| Diabetes | 1.39 (1.19-1.62) | <0.01 |
| Hypertension | 1.32 (1.12-1.54) | <0.01 |
| Heart failure | 1.59 (1.27-2.00) | <0.01 |
| Coronary artery disease | 1.45 (1.23-1.72) | <0.01 |
| Peripheral Vascular Disease | 2.16 (1.76-2.54) | <0.01 |
| Previous stroke or transient ischaemic attack | 1.43 (1.18-1.74) | <0.01 |
| Chronic Obstructive Pulmonary Disease | 1.22 (1.00-1.49) | 0.05 |
| eGFR |  |  |
| <30 | 10.47 (8.47-12.94) | <0.01 |
| 30-45 | 2.45 (1.97-3.03) | <0.01 |
| 45-60 | 1.68 (1.38-2.03) | <0.01 |
| >60 | ref | ref |
| Neurosurgery | 1.13 (0.86-1.49) | 0.38 |
| Urgent or emergency surgery | 1.89 (1.60-2.23) | <0.01 |
| Major Surgery | 1.63 (1.40-1.91) | <0.01 |
| Duration of heart rate >100 bpm |  |  |
| <5 minutes | 0.92 (0.67-1.25) | 0.58 |
| 5 - 10 mintues | 0.93 (0.62-1.38) | 0.71 |
| 11 - 30 minutes | 1.07 (0.79-1.46) | 0.66 |
| >30 minutes | 2.22 (1.71-2.88) | *<0.01* |
|  |  |  |

**Supplementary table 4. Full adjusted logistic regression model for duration of intraoperative heart rate <55 beats per minute.**

**Multivariable logistic regression analysis. Dependent variables is myocardial injury within 30 days of surgery. Duration of heart rate <55 beats per minute (bpm) was stratified into quartiles. The reference group was participants with heart rate** ≥ 55 **beats per minute. Estimated glomerular filtration rate (eGFR). A post-hoc Bonferroni correction was made for the four categories of duration, where p-values should be less than 0.05/4 = 0.0125 in order to be statistically significant (indicated in italics).**

| **Covariates** | **odds ratio** | **p-value** |
| --- | --- | --- |
| Age |  |  |
| 45-64 | ref | ref |
| 65-75 | 1.09 (0.91-1.31) | 0.35 |
| >75 | 2.05 (1.72-2.44) | <0.01 |
| Male sex | 1.39 (1.21-1.59) | <0.01 |
| Comorbid disease |  |  |
| Existing Atrial Fibrillation | 1.59 (1.23-2.07) | <0.01 |
| Diabetes | 1.39 (1.20-1.62) | <0.01 |
| Hypertension | 1.31 (1.12-1.54) | <0.01 |
| Heart failure | 1.57 (1.25-1.98) | <0.01 |
| Coronary artery disease | 1.46 (1.24-1.73) | <0.01 |
| Peripheral Vascular Disease | 2.17 (1.77-2.66) | <0.01 |
| Previous stroke or transient ischaemic attack | 1.46 (1.20-1.77) | <0.01 |
| Chronic Obstructive Pulmonary Disease | 1.18 (0.97-1.45) | 0.10 |
| eGFR |  |  |
| <30 | 10.70 (8.66-13.22) | <0.01 |
| 30-45 | 2.54 (2.05-3.15) | <0.01 |
| 45-60 | 1.70 (1.40-2.05) | <0.01 |
| >60 | ref | ref |
| Neurosurgery | 1.17 (0.89-2.05) | 0.26 |
| Urgent or emergency surgery | 1.89 (1.61-2.23) | <0.01 |
| Major Surgery | 1.66 (1.42-1.94) | <0.01 |
| Duration of heart rate <55 bpm |  |  |
| <14 minutes | 0.72 (0.53-0.98) | 0.04 |
| 14 - 30 minutes | 0.75 (0.57-1.00) | 0.05 |
| 31 - 65 minutes | 0.69 (0.51-0.95) | 0.02 |
| > 65 minutes | 0.64 (0.47-0.86) | *<0.01* |
|  |  |  |

**Supplementary table 5. Full adjusted logistic regression model for highest intraoperative systolic blood pressure**

**Multivariable logistic regression analysis. Dependent variables are myocardial injury, myocardial infarction and mortality within 30 days of surgery. Intraoperative systolic blood pressure was dichotomised according to a threshold of >160 millimeters of mercury (mmHg). Systolic blood pressure ≤160 mmHg was the reference category. Estimated glomerular filtration rate (eGFR).**

|  | **Myocardial Injury** | | **Myocardial Infarction** | | **Mortality** | |
| --- | --- | --- | --- | --- | --- | --- |
| **Covariates** | **odds ratio** | **p-value** | **odds ratio** | **p-value** | **odds ratio** | **p-value** |
| Age (years) |  |  |  |  |  |  |
| 45-64 (reference) | - | - | - | - | - | - |
| 65-75 | 1.07 (0.89-1.29) | 0.46 | 1.14 (0.85-1.52) | 0.39 | 1.59 (1.14-2.22) | <0.01 |
| >75 | 2.06 (1.72-2.47) | <0.01 | 1.82 (1.39-2.39) | <0.01 | 2.29 (1.64-3.19) | <0.01 |
| Male sex | 1.35 (1.18-1.56) | <0.01 | 1.01 (0.82-1.24) | 0.96 | 1.16 (0.91-1.49) | 0.23 |
| History of atrial fibrillation | 1.68 (1.29-2.19) | <0.01 | 1.44 (1.01-2.06) | 0.05 | 1.10 (0.68-1.78) | 0.71 |
| History of diabetes | 1.38 (1.18-1.61) | <0.01 | 1.23 (0.98-1.54) | 0.08 | 1.03 (0.77-1.39) | 0.83 |
| History of hypertension | 1.30 (1.11-1.53) | <0.01 | 1.36 (1.06-1.74) | 0.02 | 0.97 (0.73-1.27) | 0.81 |
| History of heart failure | 1.55 (1.23-1.96) | <0.01 | 1.65 (1.22-2.24) | <0.01 | 1.33 (0.88-2.02) | 0.18 |
| History of coronary artery disease | 1.44 (1.22-1.71) | <0.01 | 2.12 (1.68-2.68) | <0.01 | 0.88 (0.63-1.23) | 0.46 |
| History of peripheral vascular disease | 2.25 (1.83-2.76) | <0.01 | 2.22 (1.68-2.92) | <0.01 | 1.86 (1.29-2.68) | <0.01 |
| History of stroke or transient ischaemic attack | 1.46 (1.20-1.78) | <0.01 | 1.14 (0.86-1.52) | 0.37 | 1.60 (1.14-2.24) | <0.01 |
| History of chronic obstructive pulmonary disease | 1.22 (0.99-1.49) | 0.06 | 1.19 (0.89-1.59) | 0.25 | 1.93 (1.41-2.66) | <0.01 |
| Preoperative eGFR (ml/min) |  |  |  |  |  |  |
| <30 | 10.76 (8.69-13.32) | <0.01 | 4.01 (2.97-5.41) | <0.01 | 3.25 (2.23-4.75) | <0.01 |
| 30-44 | 2.51 (2.02-3.12) | <0.01 | 1.75 (1.27-2.42) | <0.01 | 1.70 (1.14-2.53) | <0.01 |
| 45-60 | 1.63 (1.34-1.97) | <0.01 | 1.39 (1.03-1.86) | 0.03 | 0.92 (0.62-1.38) | 0.70 |
| >60 (reference) | - | - | - | - | - | - |
| Neurosurgery | 1.13 (0.86-1.50) | 0.38 | 0.59 (0.35-0.99) | 0.05 | 1.67 (1.08-2.59) | 0.02 |
| Urgent or emergency surgery | 1.99 (1.69-2.34) | <0.01 | 2.35 (1.88-2.95) | <0.01 | 3.95 (3.08-5.07) | <0.01 |
| Major Surgery | 1.67 (1.43-1.95) | <0.01 | 2.16 (1.69-2.77) | <0.01 | 1.66 (1.24-2.22) | <0.01 |
| Maximum intraoperative systolic blood pressure (mmHg) |  |  |  |  |  |  |
| >160 | 1.16 (1.01-1.34) | 0.04 | 1.34 (1.09-1.64) | 0.01 | 0.76 (0.58-0.99) | 0.04 |
|  |  |  |  |  |  |  |

**Supplementary table 6. Full adjusted logistic regression model for lowest intraoperative systolic blood pressure**

**Multivariable logistic regression analysis. Dependent variables are myocardial injury, myocardial infarction and mortality within 30 days of surgery. Intraoperative systolic blood pressure was dichotomised according to a threshold of <100 millimeters of mercury (mmHg). Systolic blood pressure ≥100 mmHg was the reference category. Estimated glomerular filtration rate (eGFR).**

|  | **Myocardial Injury** | | **Myocardial Infarction** | | **Mortality** | |
| --- | --- | --- | --- | --- | --- | --- |
| **Covariates** | **odds ratio** | **p-value** | **odds ratio** | **p-value** | **odds ratio** | **p-value** |
| Age (years) |  |  |  |  |  |  |
| 45-64 (reference) | - | - | - | - | - | - |
| 65-75 | 1.09 (0.91-1.32) | 0.36 | 1.16 (0.87-1.55) | 0.32 | 1.62 (1.16-2.26) | 0.01 |
| >75 | 2.13 (1.78-2.55) | <0.01 | 1.91 (1.45-2.50) | <0.01 | 2.39 (1.71-3.33) | <0.01 |
| Male sex | 1.35 (1.17-1.55) | <0.01 | 1.00 (0.81-1.23) | 0.99 | 1.19 (0.93-1.52) | 0.17 |
| History of atrial fibrillation | 1.68 (1.29-2.19) | <0.01 | 1.41 (0.98-2.02) | 0.06 | 1.19 (0.74-1.91) | 0.48 |
| History of diabetes | 1.40 (1.20-1.63) | <0.01 | 1.23 (0.98-1.55) | 0.07 | 1.04 (0.78-1.40) | 0.78 |
| History of hypertension | 1.32 (1.12-1.55) | <0.01 | 1.40 (1.09-1.80) | 0.01 | 0.97 (0.73-1.27) | 0.97 |
| History of heart failure | 1.56 (1.24-1.97) | <0.01 | 1.65 (1.22-2.23) | <0.01 | 1.38 (0.91-2.09) | 0.13 |
| History of coronary artery disease | 1.45 (1.22-1.72) | <0.01 | 2.13 (1.69-2.69) | <0.01 | 0.91 (0.66-1.27) | 0.59 |
| History of peripheral vascular disease | 2.29 (1.87-2.81) | <0.01 | 2.25 (1.70-2.97) | <0.01 | 1.91 (1.32-2.75) | <0.01 |
| History of stroke or transient ischaemic attack | 1.48 (1.21-1.80) | <0.01 | 1.17 (0.88-1.56) | 0.29 | 1.57 (1.12-2.20) | 0.01 |
| History of chronic obstructive pulmonary disease | 1.20 (0.98-1.47) | 0.08 | 1.15 (0.86-1.54) | 0.35 | 1.92 (1.40-2.64) | <0.01 |
| Preoperative eGFR (ml/min) |  |  |  |  |  |  |
| <30 | 10.82 (8.74-13.40) | <0.01 | 4.00 (2.96-5.39) | <0.01 | 3.26 (2.23-4.76) | <0.01 |
| 30-44 | 2.52 (2.03-3.13) | <0.01 | 1.76 (1.28-2.43) | <0.01 | 1.73 (1.17-2.57) | 0.01 |
| 45-60 | 1.63 (1.34-1.98) | <0.01 | 1.39 (1.04-1.87) | 0.03 | 0.91 (0.61-1.37) | 0.66 |
| >60 (reference) | - | - | - | - | - | - |
| Neurosurgery | 1.13 (0.86-1.50) | 0.38 | 0.60 (0.36-1.00) | 0.05 | 1.58 (1.02-2.44) | 0.04 |
| Urgent or emergency surgery | 1.99 (1.69-2.34) | <0.01 | 2.35 (1.88-2.95) | <0.01 | 3.89 (3.04-4.99) | <0.01 |
| Major Surgery | 1.66 (1.42-1.94) | <0.01 | 2.15 (1.67-2.75) | <0.01 | 1.61 (1.20-2.16) | <0.01 |
| Minimum intraoperative systolic blood pressure (mmHg) |  |  |  |  |  |  |
| <100 | 1.21 (1.05-1.39) | 0.01 | 1.21 (0.98-1.49) | 0.07 | 1.81 (1.39-2.37) | <0.01 |
|  |  |  |  |  |  |  |

**Supplementary table 7. Full adjusted logistic regression model for duration of intraoperative systolic blood pressure >160 mmHg.**

**Multivariable logistic regression analysis. Dependent variable is myocardial injury within 30 days of surgery. Duration of systolic blood pressure (SBP) >160 mmHg was stratified into quartiles. The reference group was participants with SBP ≤160 mmHg. Estimated glomerular filtration rate (eGFR). A post-hoc Bonferroni correction was made for the four categories of duration, where p-values should be less than 0.05/4 = 0.0125 in order to be statistically significant (indicated in italics).**

| **Covariates** | **odds ratio** | **p-value** |
| --- | --- | --- |
| Age |  |  |
| 45-64 | ref | ref |
| 65-75 | 1.07 (0.89-1.29) | 0.47 |
| >75 | 2.00 (1.68-2.39) | <0.01 |
| Male sex | 1.38 (1.21-1.59) | <0.01 |
| Comorbid disease |  |  |
| Existing Atrial Fibrillation | 1.64 (1.26-2.13) | <0.01 |
| Diabetes | 1.40 (1.20-1.63) | <0.01 |
| Hypertension | 1.29 (1.10-1.51) | <0.01 |
| Heart failure | 1.59 (1.26-1.99) | <0.01 |
| Coronary artery disease | 1.43 (1.21-1.69) | <0.01 |
| Peripheral Vascular Disease | 2.15 (1.76-2.63) | <0.01 |
| Previous stroke or transient ischaemic attack | 1.44 (1.18-1.75) | <0.01 |
| Chronic Obstructive Pulmonary Disease | 1.23 (1.01-1.50) | 0.04 |
| eGFR |  |  |
| <30 | 10.87 (8.80-13.42) | <0.01 |
| 30-45 | 2.51 (2.02-3.11) | <0.01 |
| 45-60 | 1.67 (1.38-2.02) | <0.01 |
| >60 | ref | ref |
| Neurosurgery | 1.11 (0.84-1.46) | 0.48 |
| Urgent or emergency surgery | 1.98 (1.69-2.33) | <0.01 |
| Major Surgery | 1.67 (1.43-1.94) | <0.01 |
| Duration of systolic blood pressure >160 mmHg |  |  |
| <6 minutes | 1.20 (0.95-1.52) | 0.13 |
| 6 - 12 minutes | 1.19 (0.92-1.53) | 0.18 |
| 13 - 25 minutes | 1.11 (0.89-1.38) | 0.38 |
| >25 minutes | 1.16 (0.92-1.48) | 0.21 |
|  |  |  |

**Supplementary table 8. Full adjusted logistic regression model for duration of intraoperative systolic blood pressure <100 mmHg.**

**Multivariable logistic regression analysis. Dependent variable is myocardial injury within 30 days of surgery. Duration of systolic blood pressure (SBP) <100 mmHg was stratified into quartiles. The reference group was participants with SBP ≥100 mmHg. Estimated glomerular filtration rate (eGFR). A post-hoc Bonferroni correction was made for the four categories of duration, where p-values should be less than 0.05/4 = 0.0125 in order to be statistically significant (indicated in italics).**

| **Covariates** | **odds ratio** | **p-value** |
| --- | --- | --- |
| Age |  |  |
| 45-64 | ref | ref |
| 65-75 | 1.10 (0.91-1.32) | 0.34 |
| >75 | 2.09 (1.75-2.50) | <0.01 |
| Male sex | 1.38 (1.20-1.58) | <0.01 |
| Comorbid disease |  |  |
| Existing Atrial Fibrillation | 1.63 (1.25-2.12) | <0.01 |
| Diabetes | 1.41 (1.21-1.64) | <0.01 |
| Hypertension | 1.31 (1.12-1.53) | <0.01 |
| Heart failure | 1.58 (1.26-1.99) | <0.01 |
| Coronary artery disease | 1.44 (1.22-1.71) | <0.01 |
| Peripheral Vascular Disease | 2.18 (1.78-2.67) | <0.01 |
| Previous stroke or transient ischaemic attack | 1.47 (1.21-1.78) | <0.01 |
| Chronic Obstructive Pulmonary Disease | 1.21 (0.99-1.u48) | 0.06 |
| eGFR |  |  |
| <30 | 10.92 (8.84-13.49) | <0.01 |
| 30-45 | 2.52 (2.03-3.12) | <0.01 |
| 45-60 | 1.67 (1.38-2.03) | <0.01 |
| >60 | ref | ref |
| Neurosurgery | 1.10 (0.83-1.45) | 0.50 |
| Urgent or emergency surgery | 1.99 (1.70-2.35) | <0.01 |
| Major Surgery | 1.64 (1.41-1.91) | <0.01 |
| Duration of systolic blood pressure <100 mmHg |  |  |
| <15 minutes | 1.26 (1.04-1.54) | 0.02 |
| 15 - 30 minutes | 1.10 (0.91-1.33) | 0.33 |
| 31 - 61 minutes | 1.05 (0.84-1.30) | 0.69 |
| >61 minutes | 1.33 (1.08-1.64) | *<0.01* |
|  |  |  |

**Supplementary table 9. Multivariable logistic regression model for combinations of high intraoperative systolic blood pressure and high heart rate**

**The dependent variable was myocardial injury within 30 days after surgery. The sample was categorized according to highest intraoperative systolic blood pressure (SBP) >160 mmHg, and highest intraoperative heart rate (HR) >100 beats per minute (bpm). The reference group was SBP ≤160mmHg and heart rate ≤100 bpm. The analysis was corrected for potentially confounding factors; estimated glomerular filtration rate (eGFR). Results presented as odds ratios with 95% confidence intervals (95% CI) with lower and upper bounds shown. Test for interaction between maximum SBP >160 mmHg and minimum HR (continuous) was p = 0.85 and maximum SBP (continuous) and minimum HR >100 bpm was p = 0.09.**

|  | **Myocardial Injury** | | | |
| --- | --- | --- | --- | --- |
|  |  | **95% CI** | |  |
| **Covariates** | **odds ratio** | **Lower** | **Upper** | **p-value** |
| Age (years) |  |  |  |  |
| 45-64 (reference) | - | - | - | - |
| 65-75 | 1.09 | 0.90 | 1.31 | 0.39 |
| >75 | 2.09 | 1.75 | 2.50 | 0.00 |
| Male sex | 1.35 | 1.18 | 1.55 | 0.00 |
| Comorbid disease |  |  |  |  |
| Existing Atrial Fibrillation | 1.65 | 1.27 | 2.15 | 0.00 |
| Diabetes | 1.37 | 1.17 | 1.60 | 0.00 |
| Hypertension | 1.31 | 1.12 | 1.54 | 0.00 |
| Heart failure | 1.57 | 1.24 | 1.97 | 0.00 |
| Coronary artery disease | 1.46 | 1.23 | 1.73 | 0.00 |
| Peripheral Vascular Disease | 2.27 | 1.85 | 2.78 | 0.00 |
| Previous stroke or transient ischaemic attack | 1.45 | 1.19 | 1.76 | 0.00 |
| Chronic Obstructive Pulmonary Disease | 1.22 | 1.00 | 1.50 | 0.05 |
| eGFR (ml/min) |  |  |  |  |
| <30 | 10.59 | 8.55 | 13.11 | 0.00 |
| 30-45 | 2.49 | 2.01 | 3.10 | 0.00 |
| 45-60 | 1.63 | 1.34 | 1.98 | 0.00 |
| >60 (reference) | - | - | - | - |
| Neurosurgery | 1.14 | 0.86 | 1.51 | 0.36 |
| Urgent or emergency surgery | 1.94 | 1.65 | 2.29 | 0.00 |
| Major Surgery | 1.66 | 1.42 | 1.95 | 0.00 |
| Maximum SBP and maximum HR |  |  |  |  |
| SBP ≤160 & HR ≤100 (reference) | - | - | - | - |
| SBP >160 & HR ≤100 | 1.22 | 1.04 | 1.43 | 0.02 |
| SBP ≤160 & HR >100 | 1.40 | 1.11 | 1.75 | 0.00 |
| SBP >160 & HR >100 | 1.23 | 0.98 | 1.54 | 0.07 |
|  |  |  |  |  |

**Supplementary table 10. Multivariable logistic regression model for combinations of low intraoperative systolic blood pressure and high heart rate**

**The dependent variable was myocardial injury within 30 days after surgery. The sample was categorized according to lowest intraoperative systolic blood pressure (SBP) <100 mmHg, and highest intraoperative heart rate (HR) >100 beats per minute (bpm). The reference group was SBP ≥100mmHg and heart rate ≤100 bpm. The analysis was corrected for potentially confounding factors; estimated glomerular filtration rate (eGFR). Results presented as odds ratios with 95% confidence intervals (95% CI) with lower and upper bounds shown. Test for interaction between minimum SBP <100 mmHg and maximum HR (continuous) was p = 0.18 and minimum SBP (continuous) and maximum HR >100 bpm was p = 0.38.**

|  | **Myocardial Injury** | | | |
| --- | --- | --- | --- | --- |
|  |  | **95% CI** | |  |
| **Covariates** | **odds ratio** | **Lower** | **Upper** | **p-value** |
| Age (years) |  |  |  |  |
| 45-64 (reference) | - | - | - | - |
| 65-75 | 1.10 | 0.92 | 1.33 | 0.30 |
| >75 | 2.16 | 1.80 | 2.58 | 0.00 |
| Male sex | 1.35 | 1.17 | 1.55 | 0.00 |
| Comorbid disease |  |  |  |  |
| Existing Atrial Fibrillation | 1.63 | 1.25 | 2.13 | 0.00 |
| Diabetes | 1.38 | 1.19 | 1.61 | 0.00 |
| Hypertension | 1.33 | 1.13 | 1.56 | 0.00 |
| Heart failure | 1.57 | 1.24 | 1.97 | 0.00 |
| Coronary artery disease | 1.47 | 1.24 | 1.74 | 0.00 |
| Peripheral Vascular Disease | 2.30 | 1.88 | 2.83 | 0.00 |
| Previous stroke or transient ischaemic attack | 1.47 | 1.21 | 1.79 | 0.00 |
| Chronic Obstructive Pulmonary Disease | 1.21 | 0.98 | 1.48 | 0.07 |
| eGFR (ml/min) |  |  |  |  |
| <30 | 10.70 | 8.64 | 13.25 | 0.00 |
| 30-45 | 2.52 | 2.02 | 3.13 | 0.00 |
| 45-60 | 1.64 | 1.35 | 1.99 | 0.00 |
| >60 (reference) | - | - | - | - |
| Neurosurgery | 1.14 | 0.86 | 1.51 | 0.37 |
| Urgent or emergency surgery | 1.95 | 1.65 | 2.29 | 0.00 |
| Major Surgery | 1.65 | 1.41 | 1.93 | 0.00 |
| Minimum SBP and maximum HR |  |  |  |  |
| SBP ≥100 & HR ≤100 (reference) | - | - | - | - |
| SBP <100 & HR ≤100 | 1.20 | 1.03 | 1.40 | 0.02 |
| SBP ≥100 & HR >100 | 1.26 | 0.94 | 1.69 | 0.13 |
| SBP <100 & HR >100 | 1.42 | 1.15 | 1.76 | 0.00 |
|  |  |  |  |  |

**Supplementary table 11. Multivariable logistic regression model for combinations of high intraoperative systolic blood pressure and low heart rate**

**The dependent variable was myocardial injury within 30 days after surgery. The sample was categorized according to highest intraoperative systolic blood pressure (SBP) >160 mmHg, and lowest intraoperative heart rate (HR) <55 beats per minute (bpm). The reference group was SBP ≤160mmHg and heart rate ≥55 bpm. The analysis was corrected for potentially confounding factors; estimated glomerular filtration rate (eGFR). Results presented as odds ratios with 95% confidence intervals (95% CI) with lower and upper bounds shown. Test for interaction between maximum SBP >160 mmHg and minimum HR (continuous) was p = 0.25 and maximum SBP (continuous) and minimum HR <55 bpm was p = 0.41.**

|  | **Myocardial Injury** | | | |
| --- | --- | --- | --- | --- |
|  |  | **95% CI** | |  |
| **Covariates** | **odds ratio** | **Lower** | **Upper** | **p-value** |
| Age (years) |  |  |  |  |
| 45-64 (reference) | - | - | - | - |
| 65-75 | 1.09 | 0.90 | 1.31 | 0.38 |
| >75 | 2.08 | 1.74 | 2.49 | 0.00 |
| Male sex | 1.37 | 1.19 | 1.57 | 0.00 |
| Comorbid disease |  |  |  |  |
| Existing Atrial Fibrillation | 1.65 | 1.27 | 2.15 | 0.00 |
| Diabetes | 1.36 | 1.17 | 1.59 | 0.00 |
| Hypertension | 1.31 | 1.12 | 1.54 | 0.00 |
| Heart failure | 1.55 | 1.23 | 1.95 | 0.00 |
| Coronary artery disease | 1.47 | 1.24 | 1.75 | 0.00 |
| Peripheral Vascular Disease | 2.27 | 1.85 | 2.79 | 0.00 |
| Previous stroke or transient ischaemic attack | 1.46 | 1.20 | 1.77 | 0.00 |
| Chronic Obstructive Pulmonary Disease | 1.18 | 0.96 | 1.45 | 0.11 |
| eGFR (ml/min) |  |  |  |  |
| <30 | 10.61 | 8.57 | 13.14 | 0.00 |
| 30-45 | 2.52 | 2.03 | 3.13 | 0.00 |
| 45-60 | 1.64 | 1.35 | 1.99 | 0.00 |
| >60 (reference) | - | - | - | - |
| Neurosurgery | 1.18 | 0.89 | 1.57 | 0.24 |
| Urgent or emergency surgery | 1.88 | 1.59 | 2.22 | 0.00 |
| Major Surgery | 1.66 | 1.42 | 1.94 | 0.00 |
| Maximum SBP and minimum HR |  |  |  |  |
| SBP ≤160 & HR ≥55 (reference) | - | - | - | - |
| SBP >160 & HR ≥55 | 1.22 | 1.04 | 1.43 | 0.02 |
| SBP ≤160 & HR <55 | 0.70 | 0.56 | 0.87 | 0.00 |
| SBP >160 & HR <55 | 0.78 | 0.61 | 0.99 | 0.05 |
|  |  |  |  |  |

**Supplementary table 12. Multivariable logistic regression model for combinations of low intraoperative systolic blood pressure and low heart rate**

**The dependent variable was myocardial injury within 30 days after surgery. The sample was categorized according to highest intraoperative systolic blood pressure (SBP) <100 mmHg, and lowest intraoperative heart rate (HR) <55 beats per minute (bpm). The reference group was SBP ≥100mmHg and heart rate ≥55 bpm. The analysis was corrected for potentially confounding factors; estimated glomerular filtration rate (eGFR). Results presented as odds ratios with 95% confidence intervals (95% CI) with lower and upper bounds shown. Test for interaction between minimum SBP <100 mmHg and minimum HR (continuous) was p = 0.11 and minimum SBP (continuous) and minimum HR <55 bpm was p = 0.79.**

|  | **Myocardial Injury** | | | |
| --- | --- | --- | --- | --- |
|  |  | **95% CI** | |  |
| **Covariates** | **odds ratio** | **Lower** | **Upper** | **p-value** |
| Age (years) |  |  |  |  |
| 45-64 (reference) | - | - | - | - |
| 65-75 | 1.11 | 0.92 | 1.34 | 0.26 |
| >75 | 2.17 | 1.82 | 2.60 | 0.00 |
| Male sex | 1.36 | 1.18 | 1.56 | 0.00 |
| Comorbid disease |  |  |  |  |
| Existing Atrial Fibrillation | 1.64 | 1.26 | 2.13 | 0.00 |
| Diabetes | 1.38 | 1.18 | 1.61 | 0.00 |
| Hypertension | 1.33 | 1.13 | 1.56 | 0.00 |
| Heart failure | 1.55 | 1.23 | 1.95 | 0.00 |
| Coronary artery disease | 1.49 | 1.25 | 1.77 | 0.00 |
| Peripheral Vascular Disease | 2.32 | 1.89 | 2.85 | 0.00 |
| Previous stroke or transient ischaemic attack | 1.49 | 1.23 | 1.82 | 0.00 |
| Chronic Obstructive Pulmonary Disease | 1.16 | 0.95 | 1.42 | 0.15 |
| eGFR (ml/min) |  |  |  |  |
| <30 | 10.65 | 8.60 | 13.20 | 0.00 |
| 30-45 | 2.55 | 2.05 | 3.17 | 0.00 |
| 45-60 | 1.65 | 1.36 | 2.00 | 0.00 |
| >60 (reference) | - | - | - | - |
| Neurosurgery | 1.19 | 0.90 | 1.58 | 0.22 |
| Urgent or emergency surgery | 1.88 | 1.59 | 2.22 | 0.00 |
| Major Surgery | 1.64 | 1.40 | 1.92 | 0.00 |
| Maximum SBP and maximum HR |  |  |  |  |
| SBP ≥100 & HR ≥55 (reference) | - | - | - | - |
| SBP <100 & HR ≥55 | 1.22 | 1.04 | 1.42 | 0.02 |
| SBP ≥100 & HR <55 | 0.60 | 0.44 | 0.81 | 0.00 |
| SBP <100 & HR <55 | 0.85 | 0.69 | 1.05 | 0.13 |

**Supplementary table 13. Sensitivity analysis excluding participants undergoing emergency surgery.**

**Multivariable logistic regression models for highest intraoperative heart rate. Dependent variables are myocardial injury, myocardial infarction and mortality within 30 days of surgery. Intraoperative heart rate was dichotomised according to a threshold of >100 beat per minute (bpm). Heart rate ≤100 bpm was the reference category. Estimated glomerular filtration rate (eGFR).**

|  | **Myocardial Injury** | | | | **Myocardial Infarction** | | | | **Mortality** | | | |
| --- | --- | --- | --- | --- | --- | --- | --- | --- | --- | --- | --- | --- |
|  |  | **95% CI** | |  |  | **95% CI** | |  |  | **95% CI** | |  |
| **Covariates** | **odds ratio** | **Lower** | **Upper** | **p-value** | **odds ratio** | **Lower** | **Upper** | **p-value** | **odds ratio** | **Lower** | **Upper** | **p-value** |
| Age |  |  |  |  |  |  |  |  |  |  |  |  |
| 45-64 (reference) | - | - | - | - | - | - | - | - | - | - | - | - |
| 65-75 | 1.10 | 0.90 | 1.34 | 0.35 | 1.22 | 0.88 | 1.68 | 0.23 | 1.69 | 1.15 | 2.49 | 0.01 |
| >75 | 2.08 | 1.72 | 2.53 | <0.01 | 2.24 | 1.65 | 3.04 | <0.01 | 2.52 | 1.70 | 3.73 | <0.01 |
| Male sex | 1.38 | 1.19 | 1.61 | <0.01 | 1.05 | 0.83 | 1.32 | 0.70 | 1.24 | 0.92 | 1.67 | 0.16 |
| Comorbid disease |  |  |  |  |  |  |  |  |  |  |  |  |
| History of atrial fibrillation | 1.93 | 1.46 | 2.55 | <0.01 | 1.51 | 1.02 | 2.25 | 0.04 | 1.16 | 0.67 | 2.03 | 0.60 |
| History of diabetes | 1.34 | 1.13 | 1.59 | <0.01 | 1.17 | 0.90 | 1.51 | 0.24 | 0.90 | 0.63 | 1.28 | 0.55 |
| History of hypertension | 1.24 | 1.04 | 1.48 | 0.02 | 1.24 | 0.94 | 1.64 | 0.13 | 1.03 | 0.74 | 1.44 | 0.85 |
| History of heart failure | 1.47 | 1.14 | 1.89 | <0.01 | 1.47 | 1.04 | 2.08 | 0.03 | 1.14 | 0.68 | 1.91 | 0.63 |
| History of coronary artery disease | 1.53 | 1.28 | 1.84 | <0.01 | 2.38 | 1.84 | 3.09 | <0.01 | 1.14 | 0.77 | 1.67 | 0.51 |
| History of peripheral vascular disease | 2.04 | 1.63 | 2.55 | <0.01 | 2.14 | 1.56 | 2.92 | <0.01 | 1.49 | 0.94 | 2.38 | 0.09 |
| History of stroke or transient ischaemic attack | 1.47 | 1.19 | 1.83 | <0.01 | 1.13 | 0.81 | 1.57 | 0.48 | 1.68 | 1.14 | 2.49 | 0.01 |
| History of chronic obstructive pulmonary disease | 1.28 | 1.03 | 1.59 | 0.03 | 1.12 | 0.81 | 1.56 | 0.48 | 2.33 | 1.63 | 3.35 | <0.01 |
| Preoperative eGFR (ml/min) |  |  |  |  |  |  |  |  |  |  |  |  |
| <30 | 11.84 | 9.37 | 14.98 | <0.01 | 4.11 | 2.90 | 5.82 | <0.01 | 2.26 | 1.35 | 3.80 | <0.01 |
| 30-44 | 2.68 | 2.11 | 3.41 | <0.01 | 1.72 | 1.19 | 2.49 | <0.01 | 1.56 | 0.96 | 2.52 | 0.07 |
| 45-60 | 1.65 | 1.34 | 2.05 | <0.01 | 1.41 | 1.01 | 1.95 | 0.04 | 0.83 | 0.51 | 1.35 | 0.46 |
| >60 (reference) | - | - | - | - | - | - | - | - | - | - | - | - |
| Neurosurgery | 1.11 | 0.82 | 1.48 | 0.51 | 0.56 | 0.32 | 0.97 | 0.04 | 1.77 | 1.09 | 2.87 | 0.02 |
| Urgent or emergency surgery | 2.12 | 1.54 | 2.92 | <0.01 | 2.58 | 1.70 | 3.90 | <0.01 | 3.87 | 2.54 | 5.90 | <0.01 |
| Major Surgery | 1.70 | 1.44 | 2.01 | <0.01 | 2.48 | 1.87 | 3.29 | <0.01 | 1.30 | 0.94 | 1.80 | 0.12 |
| Maximum intraoperative heart rate (bpm) |  |  |  |  |  |  |  |  |  |  |  |  |
| >100 | 1.38 | 1.14 | 1.66 | <0.01 | 1.46 | 1.10 | 1.93 | 0.01 | 2.82 | 2.08 | 3.82 | <0.01 |
|  |  |  |  |  |  |  |  |  |  |  |  |  |

**Supplementary table 14. Sensitivity analysis excluding participants undergoing emergency surgery.**

**Multivariable logistic regression models for lowest intraoperative heart rate. Dependent variables are myocardial injury, myocardial infarction and mortality within 30 days of surgery. Intraoperative heart rate was dichotomised according to a threshold of <55 beat per minute (bpm). Heart rate ≥55 bpm was the reference category. Estimated glomerular filtration rate (eGFR).**

|  | **Myocardial Injury** | | | | **Myocardial Infarction** | | | | **Mortality** | | | |
| --- | --- | --- | --- | --- | --- | --- | --- | --- | --- | --- | --- | --- |
|  |  | **95% CI** | |  |  | **95% CI** | |  |  | **95% CI** | |  |
| **Covariates** | **odds ratio** | **Lower** | **Upper** | **p-value** | **odds ratio** | **Lower** | **Upper** | **p-value** | **odds ratio** | **Lower** | **Upper** | **p-value** |
| Age |  |  |  |  |  |  |  |  |  |  |  |  |
| 45-64 (reference) | - | - | - | - | - | - | - | - | - | - | - | - |
| 65-75 | 1.10 | 0.90 | 1.34 | 0.35 | 1.21 | 0.88 | 1.67 | 0.23 | 1.61 | 1.10 | 2.37 | 0.02 |
| >75 | 2.06 | 1.70 | 2.51 | <0.01 | 2.22 | 1.64 | 3.01 | <0.01 | 2.38 | 1.61 | 3.51 | <0.01 |
| Male sex | 1.40 | 1.20 | 1.62 | <0.01 | 1.06 | 0.84 | 1.33 | 0.65 | 1.27 | 0.95 | 1.71 | 0.11 |
| Comorbid disease |  |  |  |  |  |  |  |  |  |  |  |  |
| History of atrial fibrillation | 1.95 | 1.48 | 2.58 | <0.01 | 1.55 | 1.04 | 2.30 | 0.03 | 1.30 | 0.75 | 2.24 | 0.35 |
| History of diabetes | 1.35 | 1.14 | 1.59 | <0.01 | 1.17 | 0.91 | 1.52 | 0.23 | 0.92 | 0.64 | 1.31 | 0.64 |
| History of hypertension | 1.24 | 1.04 | 1.47 | 0.02 | 1.23 | 0.93 | 1.63 | 0.15 | 1.01 | 0.73 | 1.40 | 0.96 |
| History of heart failure | 1.44 | 1.12 | 1.85 | 0.01 | 1.44 | 1.02 | 2.04 | 0.04 | 1.08 | 0.64 | 1.80 | 0.78 |
| History of coronary artery disease | 1.53 | 1.28 | 1.84 | <0.01 | 2.35 | 1.81 | 3.04 | <0.01 | 1.06 | 0.72 | 1.55 | 0.77 |
| History of peripheral vascular disease | 2.04 | 1.63 | 2.55 | <0.01 | 2.12 | 1.55 | 2.90 | <0.01 | 1.48 | 0.94 | 2.34 | 0.09 |
| History of stroke or transient ischaemic attack | 1.51 | 1.22 | 1.87 | <0.01 | 1.16 | 0.83 | 1.61 | 0.38 | 1.87 | 1.27 | 2.75 | <0.01 |
| History of chronic obstructive pulmonary disease | 1.24 | 0.99 | 1.54 | 0.06 | 1.10 | 0.80 | 1.53 | 0.56 | 2.25 | 1.57 | 3.22 | <0.01 |
| Preoperative eGFR (ml/min) |  |  |  |  |  |  |  |  |  |  |  |  |
| <30 | 11.73 | 9.27 | 14.84 | <0.01 | 4.06 | 2.87 | 5.76 | <0.01 | 2.26 | 1.35 | 3.78 | <0.01 |
| 30-44 | 2.74 | 2.16 | 3.48 | <0.01 | 1.74 | 1.20 | 2.52 | <0.01 | 1.63 | 1.01 | 2.63 | 0.05 |
| 45-60 | 1.67 | 1.35 | 2.06 | <0.01 | 1.41 | 1.01 | 1.95 | 0.04 | 0.82 | 0.51 | 1.33 | 0.42 |
| >60 (reference) | - | - | - | - | - | - | - | - | - | - | - | - |
| Neurosurgery | 1.15 | 0.85 | 1.54 | 0.37 | 0.57 | 0.33 | 1.00 | 0.05 | 1.78 | 1.10 | 2.88 | 0.02 |
| Urgent or emergency surgery | 2.15 | 1.56 | 2.95 | <0.01 | 2.72 | 1.81 | 4.09 | <0.01 | 4.56 | 3.03 | 6.87 | <0.01 |
| Major Surgery | 1.70 | 1.44 | 2.01 | <0.01 | 2.47 | 1.86 | 3.28 | <0.01 | 1.32 | 0.95 | 1.82 | 0.10 |
| Minimum intraoperative heart rate (bpm) |  |  |  |  |  |  |  |  |  |  |  |  |
| <55 | 0.70 | 0.59 | 0.83 | <0.01 | 0.76 | 0.58 | 1.00 | <0.05 | 0.57 | 0.39 | 0.83 | <0.01 |
|  |  |  |  |  |  |  |  |  |  |  |  |  |

**Supplementary table 15. Sensitivity analysis excluding participants undergoing emergency surgery.**

**Multivariable logistic regression models for highest intraoperative systolic blood pressure. Dependent variables are myocardial injury, myocardial infarction and mortality within 30 days of surgery. Intraoperative systolic blood pressure (SBP) was dichotomised according to a threshold of >160 millimetres of mercury (mmHg). SBP ≤160 mmHg was the reference category. Estimated glomerular filtration rate (eGFR).**

|  | **Myocardial Injury** | | | | **Myocardial Infarction** | | | | **Mortality** | | | |
| --- | --- | --- | --- | --- | --- | --- | --- | --- | --- | --- | --- | --- |
|  |  | **95% CI** | |  |  | **95% CI** | |  |  | **95% CI** | |  |
| **Covariates** | **odds ratio** | **Lower** | **Upper** | **p-value** | **odds ratio** | **Lower** | **Upper** | **p-value** | **odds ratio** | **Lower** | **Upper** | **p-value** |
| Age |  |  |  |  |  |  |  |  |  |  |  |  |
| 45-64 (reference) | - | - | - | - | - | - | - | - | - | - | - | - |
| 65-75 | 1.09 | 0.89 | 1.33 | 1.18 | 1.18 | 0.86 | 1.64 | 0.31 | 1.67 | 1.13 | 2.45 | 0.01 |
| >75 | 2.09 | 1.71 | 2.54 | 2.18 | 2.18 | 1.60 | 2.97 | <0.01 | 2.45 | 1.65 | 3.62 | <0.01 |
| Male sex | 1.37 | 1.18 | 1.60 | 1.03 | 1.03 | 0.81 | 1.30 | 0.81 | 1.21 | 0.90 | 1.63 | 0.20 |
| Comorbid disease |  |  |  |  |  |  |  |  |  |  |  |  |
| History of atrial fibrillation | 2.06 | 1.55 | 2.73 | <0.01 | 1.64 | 1.10 | 2.44 | 0.02 | 1.28 | 0.73 | 2.24 | 0.39 |
| History of diabetes | 1.32 | 1.11 | 1.57 | <0.01 | 1.17 | 0.90 | 1.52 | 0.24 | 0.90 | 0.62 | 1.29 | 0.56 |
| History of hypertension | 1.23 | 1.03 | 1.47 | 0.02 | 1.21 | 0.91 | 1.61 | 0.18 | 1.01 | 0.72 | 1.41 | 0.96 |
| History of heart failure | 1.41 | 1.09 | 1.82 | 0.01 | 1.40 | 0.98 | 1.99 | 0.06 | 1.03 | 0.60 | 1.76 | 0.92 |
| History of coronary artery disease | 1.50 | 1.25 | 1.81 | <0.01 | 2.27 | 1.74 | 2.94 | <0.01 | 1.03 | 0.70 | 1.52 | 0.88 |
| History of peripheral vascular disease | 2.08 | 1.66 | 2.61 | <0.01 | 2.20 | 1.61 | 3.01 | <0.01 | 1.55 | 0.97 | 2.46 | 0.07 |
| History of stroke or transient ischaemic attack | 1.51 | 1.22 | 1.88 | <0.01 | 1.16 | 0.84 | 1.61 | 0.38 | 1.99 | 1.35 | 2.93 | <0.01 |
| History of chronic obstructive pulmonary disease | 1.28 | 1.03 | 1.59 | 0.03 | 1.17 | 0.84 | 1.62 | 0.35 | 2.20 | 1.53 | 3.18 | <0.01 |
| Preoperative eGFR (ml/min) |  |  |  |  |  |  |  |  |  |  |  |  |
| <30 | 11.86 | 9.35 | 15.05 | <0.01 | 4.15 | 2.91 | 5.90 | <0.01 | 2.39 | 1.43 | 4.01 | <0.01 |
| 30-44 | 2.68 | 2.11 | 3.41 | <0.01 | 1.75 | 1.21 | 2.54 | <0.01 | 1.58 | 0.97 | 2.58 | 0.06 |
| 45-60 | 1.58 | 1.27 | 1.96 | <0.01 | 1.35 | 0.96 | 1.88 | 0.08 | 0.74 | 0.45 | 1.23 | 0.25 |
| >60 (reference) | - | - | - | - | - | - | - | - | - | - | - | - |
| Neurosurgery | 1.12 | 0.83 | 1.51 | 0.46 | 0.58 | 0.33 | 1.02 | 0.06 | 1.74 | 1.07 | 2.81 | 0.03 |
| Urgent or emergency surgery | 2.16 | 1.57 | 2.99 | <0.01 | 2.55 | 1.67 | 3.88 | <0.01 | 4.83 | 3.19 | 7.31 | <0.01 |
| Major Surgery | 1.70 | 1.43 | 2.01 | <0.01 | 2.40 | 1.81 | 3.19 | <0.01 | 1.37 | 0.99 | 1.90 | 0.06 |
| Maximum intraoperative systolic blood pressure (mmHg) (bpm) |  |  |  |  |  |  |  |  |  |  |  |  |
| >160 | 1.22 | 1.05 | 1.43 | 0.01 | 1.40 | 1.11 | 1.77 | 0.01 | 0.78 | 0.57 | 1.08 | 0.13 |
|  |  |  |  |  |  |  |  |  |  |  |  |  |

**Supplementary table 16. Sensitivity analysis excluding participants undergoing emergency surgery.**

**Multivariable logistic regression models for lowest intraoperative systolic blood pressure. Dependent variables are myocardial injury, myocardial infarction and mortality within 30 days of surgery. Intraoperative systolic blood pressure (SBP) was dichotomised according to a threshold of <100 millimetres of mercury (mmHg). SBP ≥100 mmHg was the reference category. Estimated glomerular filtration rate (eGFR).**

|  | **Myocardial Injury** | | | | **Myocardial Infarction** | | | | **Mortality** | | | |
| --- | --- | --- | --- | --- | --- | --- | --- | --- | --- | --- | --- | --- |
|  |  | **95% CI** | |  |  | **95% CI** | |  |  | **95% CI** | |  |
| **Covariates** | **odds ratio** | **Lower** | **Upper** | **p-value** | **odds ratio** | **Lower** | **Upper** | **p-value** | **odds ratio** | **Lower** | **Upper** | **p-value** |
| Age |  |  |  |  |  |  |  |  |  |  |  |  |
| 45-64 (reference) | - | - | - | - | - | - | - | - | - | - | - | - |
| 65-75 | 1.11 | 0.91 | 1.36 | 0.30 | 1.21 | 0.88 | 1.68 | 0.24 | 1.71 | 1.16 | 2.51 | 0.01 |
| >75 | 2.19 | 1.80 | 2.67 | <0.01 | 2.33 | 1.71 | 3.17 | <0.01 | 2.59 | 1.74 | 3.84 | <0.01 |
| Male sex | 1.37 | 1.17 | 1.59 | <0.01 | 1.03 | 0.81 | 1.30 | 0.83 | 1.24 | 0.92 | 1.67 | 0.15 |
| Comorbid disease |  |  |  |  |  |  |  |  |  |  |  |  |
| History of atrial fibrillation | 2.03 | 1.54 | 2.70 | <0.01 | 1.59 | 1.07 | 2.37 | 0.02 | 1.37 | 0.79 | 2.37 | 0.27 |
| History of diabetes | 1.34 | 1.13 | 1.59 | <0.01 | 1.18 | 0.90 | 1.53 | 0.23 | 0.91 | 0.64 | 1.31 | 0.63 |
| History of hypertension | 1.25 | 1.04 | 1.49 | 0.02 | 1.25 | 0.94 | 1.66 | 0.12 | 1.00 | 0.72 | 1.39 | 0.98 |
| History of heart failure | 1.43 | 1.10 | 1.84 | 0.01 | 1.41 | 0.99 | 2.01 | 0.06 | 1.09 | 0.65 | 1.85 | 0.75 |
| History of coronary artery disease | 1.52 | 1.26 | 1.82 | <0.01 | 2.30 | 1.77 | 2.98 | <0.01 | 1.08 | 0.73 | 1.58 | 0.71 |
| History of peripheral vascular disease | 2.14 | 1.71 | 2.69 | <0.01 | 2.28 | 1.66 | 3.12 | <0.01 | 1.57 | 0.99 | 2.49 | 0.06 |
| History of stroke or transient ischaemic attack | 1.55 | 1.25 | 1.93 | <0.01 | 1.21 | 0.87 | 1.67 | 0.26 | 1.93 | 1.31 | 2.85 | <0.01 |
| History of chronic obstructive pulmonary disease | 1.27 | 1.02 | 1.58 | 0.03 | 1.13 | 0.82 | 1.57 | 0.46 | 2.21 | 1.54 | 3.18 | <0.01 |
| Preoperative eGFR (ml/min) |  |  |  |  |  |  |  |  |  |  |  |  |
| <30 | 12.02 | 9.47 | 15.25 | <0.01 | 4.15 | 2.92 | 5.91 | <0.01 | 2.47 | 1.47 | 4.14 | <0.01 |
| 30-44 | 2.69 | 2.11 | 3.42 | <0.01 | 1.76 | 1.21 | 2.55 | <0.01 | 1.61 | 1.00 | 2.60 | 0.05 |
| 45-60 | 1.60 | 1.29 | 1.98 | <0.01 | 1.36 | 0.98 | 1.90 | 0.07 | 0.74 | 0.45 | 1.23 | 0.24 |
| >60 (reference) | - | - | - | - | - | - | - | - | - | - | - | - |
| Neurosurgery | 1.12 | 0.83 | 1.51 | 0.46 | 0.59 | 0.34 | 1.03 | 0.07 | 1.70 | 1.05 | 2.75 | 0.03 |
| Urgent or emergency surgery | 2.16 | 1.56 | 2.98 | <0.01 | 2.52 | 1.65 | 3.85 | <0.01 | 4.58 | 3.02 | 6.94 | <0.01 |
| Major Surgery | 1.68 | 1.42 | 2.00 | <0.01 | 2.38 | 1.79 | 3.17 | <0.01 | 1.32 | 0.95 | 1.84 | 0.10 |
| Minimum intraoperative systolic blood pressure (mmHg) |  |  |  |  |  |  |  |  |  |  |  |  |
| <100 | 1.32 | 1.13 | 1.55 | <0.01 | 1.43 | 1.12 | 1.82 | <0.01 | 1.81 | 1.31 | 2.49 | <0.01 |
|  |  |  |  |  |  |  |  |  |  |  |  |  |

**Supplementary table 17. Sensitivity analysis excluding participants receiving beta-blockers or rate-limiting calcium channel blockers within 24 hours before surgery.**

**Multivariable logistic regression models for highest intraoperative heart rate. Dependent variables are myocardial injury, myocardial infarction and mortality within 30 days of surgery. Intraoperative heart rate was dichotomised according to a threshold of >100 beat per minute (bpm). Heart rate ≤100 bpm was the reference category. Estimated glomerular filtration rate (eGFR).**

|  | **Myocardial Injury** | | | | **Myocardial Infarction** | | | | **Mortality** | | | |
| --- | --- | --- | --- | --- | --- | --- | --- | --- | --- | --- | --- | --- |
|  |  | **95% CI** | |  |  | **95% CI** | |  |  | **95% CI** | |  |
| **Covariates** | **odds ratio** | **Lower** | **Upper** | **p-value** | **odds ratio** | **Lower** | **Upper** | **p-value** | **odds ratio** | **Lower** | **Upper** | **p-value** |
| Age |  |  |  |  |  |  |  |  |  |  |  |  |
| 45-64 (reference) | - | - | - | - | - | - | - | - | - | - | - | - |
| 65-75 | 1.12 | 0.90 | 1.39 | 0.32 | 1.09 | 0.77 | 1.54 | 0.64 | 1.68 | 1.17 | 2.41 | 0.01 |
| >75 | 2.27 | 1.85 | 2.79 | <0.01 | 1.85 | 1.34 | 2.57 | <0.01 | 2.28 | 1.59 | 3.27 | <0.01 |
| Male sex | 1.24 | 1.05 | 1.45 | 0.01 | 0.78 | 0.60 | 1.00 | 0.05 | 1.10 | 0.84 | 1.44 | 0.51 |
| Comorbid disease |  |  |  |  |  |  |  |  |  |  |  |  |
| History of atrial fibrillation | 1.79 | 1.25 | 2.55 | <0.01 | 1.28 | 0.77 | 2.12 | 0.34 | 0.81 | 0.42 | 1.57 | 0.54 |
| History of diabetes | 1.31 | 1.09 | 1.58 | 0.01 | 1.30 | 0.98 | 1.72 | 0.07 | 1.01 | 0.73 | 1.40 | 0.96 |
| History of hypertension | 1.20 | 1.01 | 1.44 | 0.04 | 1.20 | 0.91 | 1.59 | 0.21 | 1.02 | 0.76 | 1.37 | 0.91 |
| History of heart failure | 1.75 | 1.30 | 2.36 | <0.01 | 2.14 | 1.46 | 3.14 | <0.01 | 1.32 | 0.79 | 2.22 | 0.30 |
| History of coronary artery disease | 1.56 | 1.24 | 1.96 | <0.01 | 2.40 | 1.77 | 3.27 | <0.01 | 1.05 | 0.70 | 1.58 | 0.83 |
| History of peripheral vascular disease | 1.86 | 1.44 | 2.42 | <0.01 | 2.08 | 1.45 | 2.98 | <0.01 | 1.99 | 1.31 | 3.01 | <0.01 |
| History of stroke or transient ischaemic attack | 1.49 | 1.17 | 1.90 | <0.01 | 1.13 | 0.77 | 1.64 | 0.54 | 1.58 | 1.08 | 2.32 | 0.02 |
| History of chronic obstructive pulmonary disease | 1.40 | 1.11 | 1.76 | 0.01 | 1.56 | 1.12 | 2.19 | 0.01 | 2.42 | 1.72 | 3.40 | <0.01 |
| Preoperative eGFR (ml/min) |  |  |  |  |  |  |  |  |  |  |  |  |
| <30 | 12.75 | 9.86 | 16.48 | <0.01 | 4.00 | 2.73 | 5.85 | <0.01 | 3.47 | 2.25 | 5.35 | <0.01 |
| 30-44 | 2.89 | 2.23 | 3.75 | <0.01 | 1.97 | 1.32 | 2.94 | <0.01 | 1.94 | 1.25 | 3.01 | <0.01 |
| 45-60 | 1.72 | 1.36 | 2.16 | <0.01 | 1.60 | 1.13 | 2.29 | 0.01 | 1.11 | 0.72 | 1.70 | 0.64 |
| >60 (reference) | - | - | - | - | - | - | - | - | - | - | - | - |
| Neurosurgery | 1.20 | 0.87 | 1.65 | 0.27 | 0.63 | 0.34 | 1.18 | 0.15 | 1.90 | 1.20 | 3.02 | 0.01 |
| Urgent or emergency surgery | 1.91 | 1.58 | 2.30 | <0.01 | 2.53 | 1.95 | 3.30 | <0.01 | 3.69 | 2.81 | 4.85 | <0.01 |
| Major Surgery | 1.74 | 1.45 | 2.09 | <0.01 | 2.33 | 1.72 | 3.16 | <0.01 | 1.71 | 1.24 | 2.36 | <0.01 |
| Maximum intraoperative heart rate (bpm) |  |  |  |  |  |  |  |  |  |  |  |  |
| >100 | 1.28 | 1.06 | 1.55 | 0.01 | 1.46 | 1.11 | 1.93 | 0.01 | 2.23 | 1.69 | 2.94 | <0.01 |
|  |  |  |  |  |  |  |  |  |  |  |  |  |

**Supplementary table 18. Sensitivity analysis excluding participants receiving beta-blockers or rate-limiting calcium channel blockers within 24 hours before surgery.**

**Multivariable logistic regression models for lowest intraoperative heart rate. Dependent variables are myocardial injury, myocardial infarction and mortality within 30 days of surgery. Intraoperative heart rate was dichotomised according to a threshold of <55 beat per minute (bpm). Heart rate ≥55 bpm was the reference category. Estimated glomerular filtration rate (eGFR).**

|  | **Myocardial Injury** | | | | **Myocardial Infarction** | | | | **Mortality** | | | |
| --- | --- | --- | --- | --- | --- | --- | --- | --- | --- | --- | --- | --- |
|  |  | **95% CI** | |  |  | **95% CI** | |  |  | **95% CI** | |  |
| **Covariates** | **odds ratio** | **Lower** | **Upper** | **p-value** | **odds ratio** | **Lower** | **Upper** | **p-value** | **odds ratio** | **Lower** | **Upper** | **p-value** |
| Age |  |  |  |  |  |  |  |  |  |  |  |  |
| 45-64 (reference) | - | - | - | - | - | - | - | - | - | - | - | - |
| 65-75 | 1.12 | 0.90 | 1.39 | 0.32 | 1.09 | 0.77 | 1.54 | 0.63 | 1.62 | 1.13 | 2.32 | 0.01 |
| >75 | 2.26 | 1.84 | 2.77 | <0.01 | 1.83 | 1.32 | 2.54 | <0.01 | 2.16 | 1.51 | 3.09 | <0.01 |
| Male sex | 1.25 | 1.07 | 1.47 | 0.01 | 0.79 | 0.61 | 1.02 | 0.07 | 1.12 | 0.85 | 1.46 | 0.42 |
| Comorbid disease |  |  |  |  |  |  |  |  |  |  |  |  |
| History of atrial fibrillation | 1.83 | 1.28 | 2.62 | <0.01 | 1.35 | 0.82 | 2.23 | 0.24 | 0.90 | 0.47 | 1.74 | 0.76 |
| History of diabetes | 1.30 | 1.08 | 1.56 | 0.01 | 1.28 | 0.97 | 1.70 | 0.09 | 1.01 | 0.73 | 1.41 | 0.95 |
| History of hypertension | 1.19 | 1.00 | 1.42 | 0.05 | 1.18 | 0.89 | 1.56 | 0.24 | 1.00 | 0.75 | 1.35 | 0.98 |
| History of heart failure | 1.75 | 1.30 | 2.36 | <0.01 | 2.10 | 1.43 | 3.09 | <0.01 | 1.23 | 0.73 | 2.07 | 0.43 |
| History of coronary artery disease | 1.56 | 1.24 | 1.97 | <0.01 | 2.38 | 1.75 | 3.23 | <0.01 | 1.00 | 0.66 | 1.50 | 0.99 |
| History of peripheral vascular disease | 1.86 | 1.43 | 2.41 | <0.01 | 2.05 | 1.43 | 2.94 | <0.01 | 1.94 | 1.29 | 2.93 | <0.01 |
| History of stroke or transient ischaemic attack | 1.53 | 1.20 | 1.95 | <0.01 | 1.15 | 0.79 | 1.68 | 0.46 | 1.71 | 1.17 | 2.50 | 0.01 |
| History of chronic obstructive pulmonary disease | 1.36 | 1.08 | 1.71 | 0.01 | 1.51 | 1.08 | 2.12 | 0.02 | 2.34 | 1.68 | 3.28 | <0.01 |
| Preoperative eGFR (ml/min) |  |  |  |  |  |  |  |  |  |  |  |  |
| <30 | 12.67 | 9.80 | 16.39 | <0.01 | 4.03 | 2.75 | 5.88 | <0.01 | 3.72 | 2.42 | 5.71 | <0.01 |
| 30-44 | 2.92 | 2.26 | 3.79 | <0.01 | 2.01 | 1.35 | 3.00 | <0.01 | 2.05 | 1.33 | 3.18 | <0.01 |
| 45-60 | 1.72 | 1.37 | 2.17 | <0.01 | 1.60 | 1.12 | 2.28 | 0.01 | 1.09 | 0.71 | 1.68 | 0.68 |
| >60 (reference) | - | - | - | - | - | - | - | - | - | - | - | - |
| Neurosurgery | 1.26 | 0.91 | 1.73 | 0.16 | 0.66 | 0.36 | 1.24 | 0.20 | 1.89 | 1.19 | 3.00 | 0.01 |
| Urgent or emergency surgery | 1.88 | 1.56 | 2.27 | <0.01 | 2.52 | 1.94 | 3.28 | <0.01 | 3.96 | 3.02 | 5.20 | <0.01 |
| Major Surgery | 1.73 | 1.45 | 2.08 | <0.01 | 2.32 | 1.71 | 3.14 | <0.01 | 1.75 | 1.27 | 2.42 | <0.01 |
| Minimum intraoperative heart rate (bpm) |  |  |  |  |  |  |  |  |  |  |  |  |
| <55 | 0.67 | 0.54 | 0.82 | <0.01 | 0.58 | 0.41 | 0.83 | <0.01 | 0.71 | 0.49 | 1.03 | 0.07 |
|  |  |  |  |  |  |  |  |  |  |  |  |  |

**Supplementary table 19. Sensitivity analysis excluding participants with pre-existing atrial fibrillation.**

**Multivariable logistic regression models for maximum intraoperative heart rate. Dependent variables are myocardial injury, myocardial infarction and mortality within 30 days of surgery. Intraoperative heart rate was dichotomised according to a threshold of >100 beat per minute (bpm). Heart rate ≤100 bpm was the reference category. Estimated glomerular filtration rate (eGFR).**

|  | **Myocardial Injury** | | | | **Myocardial Infarction** | | | | **Mortality** | | | |
| --- | --- | --- | --- | --- | --- | --- | --- | --- | --- | --- | --- | --- |
|  |  | **95% CI** | |  |  | **95% CI** | |  |  | **95% CI** | |  |
| **Covariates** | **odds ratio** | **Lower** | **Upper** | **p-value** | **odds ratio** | **Lower** | **Upper** | **p-value** | **odds ratio** | **Lower** | **Upper** | **p-value** |
| Age |  |  |  |  |  |  |  |  |  |  |  |  |
| 45-64 (reference) | - | - | - | - | - | - | - | - | - | - | - | - |
| 65-75 | 1.09 | 0.91 | 1.32 | 0.35 | 1.20 | 0.90 | 1.61 | 0.22 | 1.67 | 1.19 | 2.36 | <0.01 |
| >75 | 2.06 | 1.72 | 2.47 | <0.01 | 1.89 | 1.43 | 2.50 | <0.01 | 2.37 | 1.69 | 3.33 | <0.01 |
| Male sex | 1.35 | 1.17 | 1.56 | <0.01 | 0.98 | 0.79 | 1.21 | 0.84 | 1.17 | 0.91 | 1.52 | 0.22 |
| Comorbid disease |  |  |  |  |  |  |  |  |  |  |  |  |
| History of diabetes | 1.46 | 1.25 | 1.71 | <0.01 | 1.28 | 1.01 | 1.62 | 0.04 | 1.04 | 0.77 | 1.41 | 0.81 |
| History of hypertension | 1.28 | 1.08 | 1.50 | <0.01 | 1.36 | 1.05 | 1.75 | 0.02 | 0.92 | 0.69 | 1.23 | 0.57 |
| History of heart failure | 1.72 | 1.34 | 2.21 | <0.01 | 1.77 | 1.28 | 2.46 | <0.01 | 1.33 | 0.83 | 2.13 | 0.24 |
| History of coronary artery disease | 1.57 | 1.31 | 1.87 | <0.01 | 2.42 | 1.90 | 3.09 | <0.01 | 1.01 | 0.71 | 1.44 | 0.96 |
| History of peripheral vascular disease | 2.09 | 1.69 | 2.60 | <0.01 | 2.03 | 1.51 | 2.74 | <0.01 | 1.74 | 1.17 | 2.59 | 0.01 |
| History of stroke or transient ischaemic attack | 1.45 | 1.18 | 1.79 | <0.01 | 0.99 | 0.72 | 1.37 | 0.96 | 1.43 | 1.00 | 2.06 | 0.05 |
| History of chronic obstructive pulmonary disease | 1.25 | 1.01 | 1.54 | 0.04 | 1.29 | 0.96 | 1.75 | 0.10 | 2.34 | 1.69 | 3.23 | <0.01 |
| Preoperative eGFR (ml/min) |  |  |  |  |  |  |  |  |  |  |  |  |
| <30 | 10.45 | 8.38 | 13.02 | <0.01 | 3.96 | 2.88 | 5.45 | <0.01 | 3.40 | 2.29 | 5.05 | <0.01 |
| 30-44 | 2.46 | 1.95 | 3.09 | <0.01 | 1.74 | 1.23 | 2.45 | <0.01 | 1.56 | 1.01 | 2.40 | 0.04 |
| 45-60 | 1.70 | 1.39 | 2.07 | <0.01 | 1.51 | 1.12 | 2.05 | 0.01 | 1.06 | 0.71 | 1.59 | 0.77 |
| >60 (reference) | - | - | - | - | - | - | - | - | - | - | - | - |
| Neurosurgery | 1.17 | 0.89 | 1.55 | 0.27 | 0.59 | 0.34 | 1.01 | 0.06 | 1.80 | 1.16 | 2.80 | 0.01 |
| Urgent or emergency surgery | 2.07 | 1.75 | 2.45 | <0.01 | 2.43 | 1.92 | 3.08 | <0.01 | 3.57 | 2.75 | 4.63 | <0.01 |
| Major Surgery | 1.63 | 1.39 | 1.91 | <0.01 | 2.18 | 1.68 | 2.84 | <0.01 | 1.59 | 1.17 | 2.15 | <0.01 |
| Maximum intraoperative heart rate (bpm) |  |  |  |  |  |  |  |  |  |  |  |  |
| >100 | 1.31 | 1.10 | 1.56 | <0.01 | 1.29 | 0.99 | 1.67 | 0.06 | 2.57 | 1.97 | 3.35 | <0.01 |
|  |  |  |  |  |  |  |  |  |  |  |  |  |

**Supplementary table 20. Sensitivity analysis excluding participants with pre-existing atrial fibrillation.**

**Multivariable logistic regression models for maximum intraoperative heart rate. Dependent variables are myocardial injury, myocardial infarction and mortality within 30 days of surgery. Intraoperative heart rate was dichotomised according to a threshold of <55 beat per minute (bpm). Heart rate ≥55 bpm was the reference category. Estimated glomerular filtration rate (eGFR).**

|  | **Myocardial Injury** | | | | **Myocardial Infarction** | | | | **Mortality** | | | |
| --- | --- | --- | --- | --- | --- | --- | --- | --- | --- | --- | --- | --- |
|  |  | **95% CI** | |  |  | **95% CI** | |  |  | **95% CI** | |  |
| **Covariates** | **odds ratio** | **Lower** | **Upper** | **p-value** | **odds ratio** | **Lower** | **Upper** | **p-value** | **odds ratio** | **Lower** | **Upper** | **p-value** |
| Age |  |  |  |  |  |  |  |  |  |  |  |  |
| 45-64 (reference) | - | - | - | - | - | - | - | - | - | - | - | - |
| 65-75 | 1.10 | 0.91 | 1.32 | 0.34 | 1.21 | 0.90 | 1.62 | 0.21 | 1.60 | 1.14 | 2.25 | 0.01 |
| >75 | 2.05 | 1.71 | 2.46 | <0.01 | 1.88 | 1.42 | 2.49 | <0.01 | 2.24 | 1.60 | 3.14 | <0.01 |
| Male sex | 1.36 | 1.18 | 1.57 | <0.01 | 0.99 | 0.80 | 1.23 | 0.92 | 1.22 | 0.95 | 1.58 | 0.13 |
| Comorbid disease |  |  |  |  |  |  |  |  |  |  |  |  |
| History of atrial fibrillation | 1.45 | 1.24 | 1.70 | <0.01 | 1.27 | 1.01 | 1.61 | 0.05 | 1.06 | 0.78 | 1.43 | 0.73 |
| History of diabetes | 1.27 | 1.08 | 1.50 | <0.01 | 1.35 | 1.04 | 1.74 | 0.02 | 0.90 | 0.68 | 1.19 | 0.45 |
| History of hypertension | 1.69 | 1.32 | 2.18 | <0.01 | 1.75 | 1.26 | 2.42 | <0.01 | 1.26 | 0.79 | 2.01 | 0.33 |
| History of heart failure | 1.58 | 1.32 | 1.88 | <0.01 | 2.42 | 1.89 | 3.08 | <0.01 | 0.94 | 0.66 | 1.33 | 0.72 |
| History of coronary artery disease | 2.08 | 1.68 | 2.58 | <0.01 | 2.01 | 1.49 | 2.72 | <0.01 | 1.69 | 1.14 | 2.50 | 0.01 |
| History of peripheral vascular disease | 1.47 | 1.19 | 1.82 | <0.01 | 1.00 | 0.72 | 1.38 | 0.98 | 1.52 | 1.06 | 2.18 | 0.02 |
| History of stroke or transient ischaemic attack | 1.21 | 0.98 | 1.49 | 0.08 | 1.26 | 0.93 | 1.71 | 0.13 | 2.22 | 1.60 | 3.06 | <0.01 |
| History of chronic obstructive pulmonary disease |  |  |  |  |  |  |  |  |  |  |  |  |
| Preoperative eGFR (ml/min) | 10.42 | 8.36 | 12.99 | <0.01 | 3.98 | 2.90 | 5.47 | <0.01 | 3.61 | 2.44 | 5.34 | <0.01 |
| <30 | 2.49 | 1.98 | 3.13 | <0.01 | 1.75 | 1.24 | 2.47 | <0.01 | 1.62 | 1.05 | 2.49 | 0.03 |
| 30-44 | 1.72 | 1.41 | 2.09 | <0.01 | 1.52 | 1.12 | 2.06 | 0.01 | 1.06 | 0.71 | 1.58 | 0.79 |
| 45-60 | 1.10 | 0.91 | 1.32 | 0.34 | 1.21 | 0.90 | 1.62 | 0.21 | 1.60 | 1.14 | 2.25 | 0.01 |
| >60 (reference) | - | - | - | - | - | - | - | - | - | - | - | - |
| Neurosurgery | 1.22 | 0.92 | 1.62 | 0.16 | 0.61 | 0.35 | 1.04 | 0.07 | 1.80 | 1.16 | 2.80 | 0.01 |
| Urgent or emergency surgery | 2.03 | 1.72 | 2.40 | <0.01 | 2.42 | 1.91 | 3.06 | <0.01 | 3.82 | 2.95 | 4.95 | <0.01 |
| Major Surgery | 1.63 | 1.39 | 1.91 | <0.01 | 2.17 | 1.67 | 2.82 | <0.01 | 1.63 | 1.20 | 2.20 | <0.01 |
| Minimum intraoperative heart rate (bpm) |  |  |  |  |  |  |  |  |  |  |  |  |
| <55 | 0.68 | 0.58 | 0.81 | <0.01 | 0.75 | 0.58 | 0.98 | 0.04 | 0.63 | 0.45 | 0.88 | 0.01 |
|  |  |  |  |  |  |  |  |  |  |  |  |  |

**Supplementary table 21. Post-hoc sensitivity analysis for four-level heart rate and systolic pressure variables: baseline data**

**Baseline data stratified by heart rate (HR) in beats per minute and systolic blood pressure (SBP) in mmHg. Estimated glomerular filtration rate (eGFR). Myocardial injury after non-cardiac surgery (MINS).**

|  | **Intraoperative heart rate (HR)** | | | | **Intraoperative systolic blood pressure (SBP)** | | | |
| --- | --- | --- | --- | --- | --- | --- | --- | --- |
|  | **HR 55-100** | **HR <55** | **HR >100** | **HR >100 & <55** | **SBP 100-160** | **SBP <100** | **SBP >160** | **SBP >160 & <100** |
| Number of cases (n) | 8022 | 3202 | 2051 | 465 | 3491 | 6081 | 1711 | 2457 |
| Mean age (SD) | 66.0 (11.8) | 66.2 (11.2) | 64.2 (12.4) | 63.6 (11.4) | 66.3 (11.7) | 63.4 (11.4) | 70.1 (11.7) | 67.3 (11.3) |
| Sex |  |  |  |  |  |  |  |  |
| Male (%) | 3838 (47.8) | 1603 (50.1) | 1024 (49.9) | 244 (52.5) | 1835 (52.6) | 2938 (48.3) | 769 (44.9) | 1167 (47.5) |
| Female (%) | 4184 (52.2) | 1599 (49.9) | 1027 (50.1) | 221 (47.5) | 1656 (47.4) | 3143 (51.7) | 942 (55.1) | 1290 (52.5) |
| Comorbid disorder (%) |  |  |  |  |  |  |  |  |
| Atrial fibrillation | 267 (3.3) | 87 (2.7) | 109 (5.3) | 12 (2.6) | 155 (4.4) | 173 (2.8) | 66 (3.9) | 81 (3.3) |
| Diabetes | 1627 (20.3) | 584 (18.2) | 477 (23.3) | 74 (15.9) | 716 (20.5) | 1019 (16.8) | 452 (26.4) | 575 (23.4) |
| Hypertension | 4221 (52.6) | 1772 (55.3) | 960 (46.8) | 185 (39.8) | 1795 (51.4) | 2817 (46.3) | 1119 (65.4) | 1407 (57.3) |
| Congestive cardiac failure | 408 (5.1) | 155 (4.8) | 91 (4.4) | 11 (2.4) | 195 (5.6) | 244 (4.0) | 107 (6.3) | 119 (4.8) |
| Coronary artery disease | 990 (12.3) | 500 (15.6) | 185 (9.0) | 34 (7.3) | 482 (13.8) | 625 (10.3) | 287 (16.8) | 315 (12.8) |
| Peripheral vascular disease | 439 (5.5) | 199 (6.2) | 98 (4.8) | 10 (2.2) | 218 (6.2) | 246 (4.0) | 152 (8.9) | 130 (5.3) |
| Previous stroke or transient ischemic attack | 522 (6.5) | 238 (7.4) | 213 (10.4) | 34 (7.3) | 234 (6.7) | 304 (5.0) | 211 (12.3) | 258 (10.5) |
| Chronic obstructive pulmonary disease (COPD) | 809 (10.1) | 223 (7.0) | 164 (8.0) | 15 (3.2) | 331 (9.5) | 524 (8.6) | 149 (8.7) | 207 (8.4) |
| Preoperative eGFR (%) |  |  |  |  |  |  |  |  |
| <30 ml/min | 304 (3.8) | 87 (2.7) | 105 (5.1) | 8 (1.7) | 130 (3.7) | 199 (3.3) | 95 (5.6) | 80 (3.3) |
| 30-45 ml/min | 407 (5.1) | 187 (5.8) | 129 (6.3) | 17 (3.7) | 167 (4.8) | 277 (4.6) | 159 (9.3) | 137 (5.6) |
| 45-60 ml/min | 852 (10.6) | 390 (12.2) | 183 (8.9) | 35 (7.5) | 372 (10.7) | 533 (8.8) | 239 (14.0) | 316 (12.9) |
| >60 ml/min | 6459 (80.5) | 2538 (79.3) | 1634 (79.7) | 405 (87.1) | 2822 (80.8) | 5072 (83.4) | 1218 (71.2) | 1924 (78.3) |
| Surgical procedure category (%) |  |  |  |  |  |  |  |  |
| Elective | 6906 (86.1) | 2964 (92.6) | 1591 (77.6) | 419 (90.1) | 3022 (86.6) | 5297 (87.1) | 1449 (84.7) | 2112 (86.0) |
| Urgent | 217 (2.7) | 56 (1.7) | 122 (5.9) | 10 (2.2) | 85 (2.4) | 175 (2.9) | 49 (2.9) | 96 (3.9) |
| Emergency | 899 (11.2) | 182 (5.7) | 338 (16.5) | 36 (7.7) | 384 (11.0) | 609 (10.0) | 213 (12.4) | 249 (10.1) |
| Major surgery (%) | 4950 (61.7) | 1951 (60.9) | 1289 (62.8) | 258 (55.5) | 1963 (56.2) | 3825 (62.9) | 1070 (62.5) | 1590 (64.7) |
| MINS (%) | 674 (8.4) | 197 (6.2) | 226 (11.0) | 20 (4.3) | 252 (7.2) | 440 (7.2) | 190 (11.1) | 235 (9.6) |

**Supplementary table 22. Post-hoc sensitivity analysis for heart rate as a four-level categorical variable. Multivariable logistic regression model. Dependent variables is myocardial injury after non-cardiac surgery (MINS). Estimated glomerular filtration rate (eGFR).**

|  | **MINS** | |
| --- | --- | --- |
| **Covariates** | **odds ratio** | **p-value** |
| Age |  |  |
| 45-64 (reference) | - | - |
| 65-75 | 1.11 (0.92-1.33) | 0.30 |
| >75 | 2.14 (1.79-2.56) | <0.01 |
| Male sex | 1.35 (1.18-1.55) | <0.01 |
| Comorbid disease |  |  |
| Existing Atrial Fibrillation | 1.60 (1.23-2.08) | <0.01 |
| Diabetes | 1.37 (1.17-1.59) | <0.01 |
| Hypertension | 1.33 (1.13-1.56) | <0.01 |
| Heart failure | 1.56 (1.23-1.96) | <0.01 |
| Coronary artery disease | 1.49 (1.25-1.76) | <0.01 |
| Peripheral Vascular Disease | 2.29 (1.86-2.81) | <0.01 |
| Previous stroke or transient ischaemic attack | 1.46 (1.20-1.78) | <0.01 |
| Chronic Obstructive Pulmonary Disease | 1.18 (0.96-1.44) | 0.11 |
| eGFR |  |  |
| <30 | 10.47 (8.45-13.00) | <0.01 |
| 30-45 | 2.52 (2.02-3.13) | <0.01 |
| 45-60 | 1.65 (1.36-2.00) | <0.01 |
| >60 (reference) | - | - |
| Neurosurgery | 1.19 (0.90-1.58) | 0.22 |
| Urgent or emergency surgery | 1.85 (1.56-2.18) | <0.01 |
| Major Surgery | 1.66 (1.42-1.94) | <0.01 |
| Intraoperative heart rate (bpm) |  |  |
| Maximum and minimum 55-100 (reference) | - | - |
| Minimum <55 | 0.73 (0.61-0.88) | <0.01 |
| Maximum >100 | 1.27 (1.06-1.52) | 0.01 |
| Minimum <55 & maximum >100 | 0.70 (0.44-1.13) | 0.15 |

**Supplementary table 23. Post-hoc sensitivity analysis for systolic blood pressure as a four-level categorical variable. Multivariable logistic regression model. Dependent variables is myocardial injury after non-cardiac surgery (MINS). Estimated glomerular filtration rate (eGFR).**

|  | **MINS** | |
| --- | --- | --- |
| **Covariates** | **odds ratio** | **p-value** |
| Age |  |  |
| 45-64 (reference) | - | - |
| 65-75 | 1.09 (0.90-1.31) | 0.38 |
| >75 | 2.11 (1.77-2.53) | <0.01 |
| Male sex | 1.36 (1.18-1.56) | <0.01 |
| Comorbid disease |  |  |
| Existing Atrial Fibrillation | 1.69 (1.30-2.20) | <0.01 |
| Diabetes | 1.38 (1.18-1.61) | <0.01 |
| Hypertension | 1.31 (1.11-1.53) | <0.01 |
| Heart failure | 1.56 (1.24-1.97) | <0.01 |
| Coronary artery disease | 1.45 (1.23-1.72) | <0.01 |
| Peripheral Vascular Disease | 2.28 (1.86-2.80) | <0.01 |
| Previous stroke or transient ischaemic attack | 1.47 (1.21-1.79) | <0.01 |
| Chronic Obstructive Pulmonary Disease | 1.21 (0.99-1.48) | 0.07 |
| eGFR |  |  |
| <30 | 10.80 (8.73-13.38) | <0.01 |
| 30-45 | 2.51 (2.02-3.12) | <0.01 |
| 45-60 | 1.62 (1.34-1.97) | <0.01 |
| >60 (reference) | - | - |
| Neurosurgery | 1.12 (0.85-1.48) | 0.42 |
| Urgent or emergency surgery | 1.99 (1.69-2.34) | <0.01 |
| Major Surgery | 1.65 (1.41-1.93) | <0.01 |
| Intraoperative systolic blood pressure (mmHg) |  |  |
| Maximum and minimum 100 - 160 (reference) | - | - |
| Minimum <100 | 1.24 (1.04-1.49) | 0.02 |
| Maximum >160 | 1.22 (0.97-1.52) | 0.08 |
| Minimum <100 & maximum >160 | 1.42 (1.16-1.75) | <0.01 |
